# Opposing roles for lipocalins and a CD36 family scavenger receptor in apical extracellular matrix-dependent protection of narrow tube integrity

**DOI:** 10.1242/dev.205309

**Published:** 2026-01-12

**Authors:** Alexandra C. Belfi, Sage G. Aviles, Rachel Forman-Rubinsky, Hasreet K. Gill, Jennifer D. Cohen, Aleksandra Nawrocka, Axelle Bourez, Pierre van Antwerpen, Patrick Laurent, Meera V. Sundaram

**Affiliations:** ^1^ Department of Genetics, University of Pennsylvania Perelman School of Medicine, Philadelphia, PA 19104, USA; ^2^ Laboratory of Neurophysiology, ULB Institute for Neuroscience, Université libre de Bruxelles, 1070 Bruxelles, Belgium; ^3^ RD3-Pharmacognosy, Bioanalysis and Drug Discovery and Analytical Platform of the Faculty of Pharmacy, Université libre de Bruxelles, 1050 Bruxelles, Belgium

**Keywords:** Apical extracellular matrix, Lipocalin, SCARB, *C. elegans*

## Abstract

All exposed epithelial surfaces, including the walls of internal tubes, are lined by a lipid and glycoprotein-rich apical extracellular matrix (aECM) that helps shape and protect the apical domain. Secreted lipocalins are lipid transporters frequently found within apical compartments. We show that loss of the *Caenorhabditis elegans* lipocalin LPR-1 disrupts the assembly of another lipocalin, LPR-3, within the pre-cuticle aECM that protects and shapes the narrow excretory duct and pore tubes. Loss of SCAV-2, a CD36 family scavenger receptor, restored LPR-3 matrix localization and suppressed the tube shaping defects of *lpr-1* and a subset of pre-cuticle mutants, but not *lpr-3* mutants. SCAV-2 accumulates at duct and pore apical surfaces and functions locally within these tubes. These data demonstrate that LPR-1 and SCAV-2 have opposing effects on narrow tube integrity by altering the content and organization of the luminal aECM of the tube, possibly by acting as transporters of LPR-3 or an LPR-3 cofactor. These results have broadly relevant implications regarding the importance of lipocalins and scavenger receptors for aECM organization and integrity of the narrowest tubes in the body.

## Teaser

**Summary:** Lipocalins and SCARBs are lipid transporters that influence susceptibility to infection and disease. This study shows that lipocalin and SCARB mutations affect the organization of a protective extracellular matrix.

## INTRODUCTION

An apical extracellular matrix (aECM) lines the environmentally-exposed apical surfaces of epithelia, such as the insides of biological tubes. Examples in humans include the vascular glycocalyx, intestinal mucin lining and lung surfactant (Gaudette et al., 2020; Johansson et al., 2013; Perez-Gil, 2008). Although the aECMs of different tissues vary in their specific contents, they all typically consist of a complex mix of proteoglycans, glycoproteins and lipoproteins or lipids that together serve to shape and protect the apical surface (Almahayni and Möckl, 2023; Zheng et al., 2020). Proteoglycans and mucins can form water-retaining gels that help expand tube lumens and protect against pathogen infection (Bulik and Robbins, 2002; Corfield, 2014; Herzog et al., 2014), while other types of aECM glycoproteins, such as zona pellucida (ZP) domain proteins, form fibrillar structures that shape the apical membrane (Bokhove and Jovine, 2018; Jovine et al., 2002; Plaza et al., 2010). The lipid components of aECM can also contribute to tube-shaping properties (Perez-Gil, 2008; Tsarouhas et al., 2023; Whitsett et al., 2015) while acting as a barrier against xenobiotics and desiccation (Feingold and Elias, 2014; Kamal et al., 2023; Njume et al., 2022; Wang et al., 2021). Defects in aECM can cause tube collapse or leakage, contributing to disorders within the pulmonary or microvascular systems (Shi et al., 2025; Toledo et al., 2025; Whitsett et al., 2015). Despite the widespread importance of aECMs for human health, we still have a limited understanding of the roles played by most individual aECM components and how different glycoproteins and lipids are apically transported and assembled to build aECMs with different functional properties.

Lipocalins and Scavenger Receptor B proteins (SCARBs) are two well-known families of lipid transporters. Lipocalins (‘fat cups’) are small, secreted, cup-shaped proteins that transport sterols, phospholipids and other hydrophobic cargoes throughout the body (Flower, 1996; Yang et al., 2023) and are most often found in apical/luminal compartments, such as mammalian plasma (di Masi et al., 2016), urine (Kuwabara et al., 2009; Lubell et al., 2022) or tear film (Dartt, 2011; Glasgow, 2021). SCARBs (including mammalian CD36, SR-B1 and LIMP2) are transmembrane proteins capable of binding and internalizing various extracellular proteins, but are best known for their roles in cholesterol and fatty acid transport (Glatz et al., 2022; Gomez-Diaz et al., 2016; Heybrock et al., 2019; Linton et al., 2017; Neculai et al., 2013). There are many reported correlations or anti-correlations between lipocalin or SCARB levels and tissue damage and disease in patient populations (Glatz et al., 2024; Koenig et al., 2021; Linton et al., 2017; Paragas et al., 2012). Potentially relevant to these observations, several recent studies in both invertebrates and mammals have suggested that lipocalins or SCARBs may affect aECM content (Feingold and Elias, 2014; Forman-Rubinsky et al., 2017; Glasgow and Abduragimov, 2021; Kook et al., 2016; Pinheiro et al., 2023; Wingen et al., 2017). Here, we report opposing roles of a lipocalin and a SCARB in aECM-dependent tube shaping in *Caenorhabditis elegans.*

**Fig. 1.**
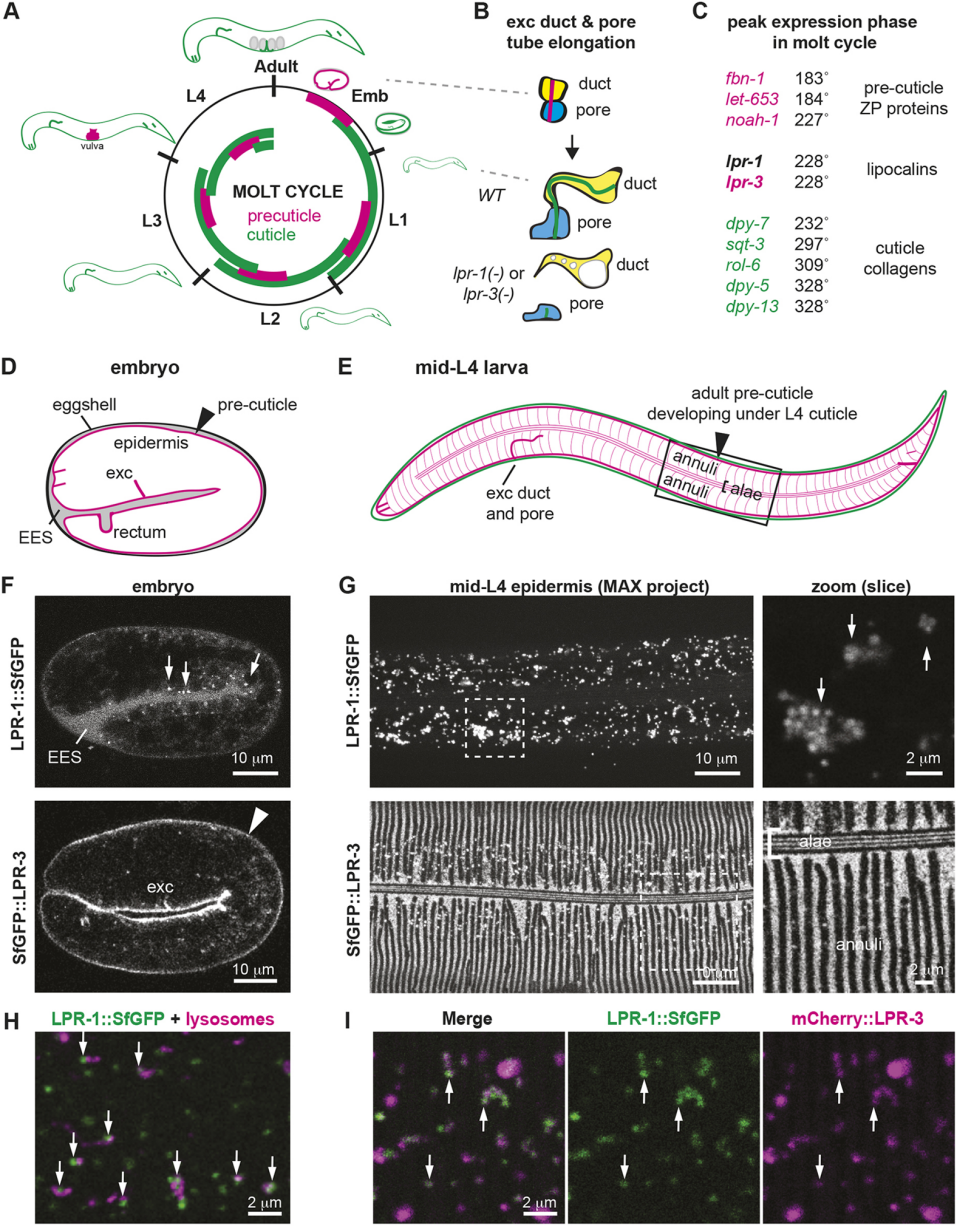
**LPR-1 does not detectably incorporate into pre-cuticle but colocalizes with LPR-3 in intracellular puncta.** (A) The *C. elegans* molt cycle. A pre-cuticle (pink) aECM precedes each cuticle (green) and then is removed by endocytosis during cuticle assembly. Adapted from Sundaram and Pujol (2024). (B) Elongation of the excretory duct and pore tubes occurs during embryogenesis, when the pre-cuticle is present. In *lpr-1* or *lpr-3* mutants, much of the lumen fragments and the remaining duct lumen swells. (C) The peak oscillatory phase of *lpr-1* expression (expressed in degrees within a 360° molt cycle) coincides with that of *lpr-3* and falls between that of known pre-cuticle components and cuticle collagens. Adapted from Meeuse et al. (2020). (D) Cartoon of 2-fold embryo, a stage when pre-cuticle is present (Sundaram and Pujol, 2024). (E) Cartoon of mid-L4 larva, when the adult pre-cuticle forms below the L4 cuticle. Box indicates mid-body region shown in confocal images below. The epidermal pre-cuticle and cuticle both contain various three-dimensional substructures, including circumferential bands (annuli) interrupted by furrows, and longitudinal ridges (alae) over the lateral epidermis (Sundaram and Pujol, 2024). (F,G) Lipocalin fusion proteins in the embryo (F) and L4 larvae (G). LPR-1 fusions are found in apical puncta (arrows) and in the extra-embryonic space (EES), whereas LPR-3 fusions mark the pre-cuticle matrix (arrowhead), including annuli and alae, along with some puncta. The number of LPR-1 (Fig. S1) and LPR-3 puncta increases with time over the molt cycle, as the pre-cuticle gets endocytosed and cleared (Birnbaum et al., 2023). (H) Many LPR-1::SfGFP puncta were found adjacent to epidermal lysosomes or LROs marked by NUC-1::mCherry (arrows). (I) LPR-1::SfGFP puncta partially overlapped with mCherry::LPR-3 puncta (arrows). Mander's coefficients: 0.72 (green+red/total green) and 0.25 (green+red/total red) at the L4.7 stage (*n*=7); the latter value is lower because many of the mCherry puncta correspond to lysosomes (see Fig. S2), where the SfGFP signal would be quenched by acidity. All other images are representative of at least *n*=10 specimens imaged. See Fig. S1 for additional images.

In the *C. elegans* embryo, external epithelia develop in the context of a pre-cuticle aECM that is eventually endocytosed and replaced by a cuticle (Birnbaum et al., 2023; Sundaram and Pujol, 2024). The pre-cuticle is required to shape tissues during morphogenesis, and the cuticle then maintains and refines that shape. The pre-cuticle is also required to properly assemble the cuticle, and these two matrices continue to alternate across the larval molt cycles, with each new pre-cuticle and cuticle forming below the older cuticle, which is then shed at the molt (Fig. 1A). The pre-cuticle is composed of chondroitin proteoglycans and various glycoproteins, including members of the ZP domain and extracellular leucine-rich repeat only (eLRRon) protein families, which are also found in many mammalian matrices (Bokhove and Jovine, 2018; Merline et al., 2009). The cuticle is composed primarily of collagens. Both matrices have a modular composition across tissues, with different subsets of components found on different regions of the epidermis and in various interfacial tubes that connect with the epidermis (Ragle et al., 2025 preprint; Sundaram and Pujol, 2024). One of the essential roles of pre-cuticle is to shape the developing excretory duct and pore tubes, which are very narrow (<0.5 μm wide) unicellular tubes that connect the excretory canal cell to the outside environment for fluid excretion (Fig. 1B) (Gill et al., 2016; Mancuso et al., 2012; Sundaram and Buechner, 2016).

Mutations in two *C. elegans* lipocalin-related proteins, LPR-1 and LPR-3, phenocopy pre-cuticle mutants to cause collapse of the excretory duct and pore tube lumens during embryo morphogenesis, leading to L1 larval lethality (Fig. 1B) (Forman-Rubinsky et al., 2017; Stone et al., 2009). Lipocalin and pre-cuticle mutants also share other matrix phenotypes such as mis-shapen cuticle ridges (alae) (Forman-Rubinsky et al., 2017; Katz et al., 2022). Whereas LPR-3 incorporates stably into the pre-cuticle, suggesting a structural role in matrix organization, LPR-1 does not detectably bind to the matrix, and it can act tissue non-autonomously when ectopically expressed in body muscle, leaving its role unclear (Pu et al., 2017; Stone et al., 2009). Despite these differences in protein localization, genetic double mutant analyses suggested that LPR-1 and LPR-3 function together in the same pathway (Forman-Rubinsky et al., 2017). Here, we show that *lpr-1* loss disrupts LPR-3 matrix incorporation, and that the lethal tube defects of *lpr-1* and some pre-cuticle mutants (but not *lpr-3* mutants) can be suppressed by loss of the SCARB SCAV-2, which restores LPR-3 to the tube matrix. These results suggest that LPR-1 and SCAV-2 have opposing effects on narrow tube integrity by altering aECM contents and organization.

## RESULTS

### LPR-1 colocalizes with LPR-3 in trafficking compartments and lysosomes

Consistent with its proposed role in pre-cuticle function, both reporter and transcriptomic data showed that *lpr-1* is most highly expressed in cuticle-producing external epithelial cells (Packer et al., 2019; Stone et al., 2009). Furthermore, we noted that, like most pre-cuticle- and cuticle-related genes, *lpr-1* mRNA expression oscillates with the molt cycle, with its peak expression phase precisely matching that of *lpr-3* and closely following that of other pre-cuticle genes and preceding that of most cuticle collagens (Fig. 1C) (Meeuse et al., 2020).

To visualize endogenous LPR-1 protein during development, we used CRISPR-Cas9 to insert fluorescent tags [Superfolder (Sf) GFP or mCherry] into the endogenous *lpr-1* locus (see Materials and Methods). These fusion proteins were functional based on >98% viability of the resulting animals (*n*>100 each). Western blotting of LPR-1::SfGFP detected a predominant band at ∼60 kd (Fig. S1A), consistent with the expected full-length tagged protein. As previously observed with either N- or C-terminally tagged transgenic fusions (Pu et al., 2017), LPR-1 fusions were apically secreted before the 2-fold stage and accumulated in closed extracellular compartments such as the space between the embryo and the eggshell (Fig. 1D,F; Fig. S1C). During its peak phase of expression at the mid-L4 larval stage, LPR-1::SfGFP marked numerous cytoplasmic puncta in the epidermis (Fig. 1F,G; Fig. S1D,E), most of which were found apically within the tissue (Figs S1B and S2A). LPR-1::SfGFP puncta did not overlap substantially with lipid droplets (Fig. S1A) but many clustered near epidermal lysosomes or lysosome-related organelles (LROs) marked by the lysosomal deoxyribonuclease NUC-1 (Fig. 1H; Fig. S2A,B). LPR-1 puncta disappeared in adults (Fig. S1D,E). Most LPR-1 puncta also contained LPR-3 at the late L4 stage, when LPR-3 is normally cleared from the pre-cuticle and endocytosed (Fig. 1I, Mander's coefficient 0.72); live imaging revealed fusion of LPR-1 and LPR-3 puncta at this stage (Movie 1; Fig. S2). Both LPR-1 and LPR-3 mCherry fusions accumulated within epidermal lysosomes marked by the SCARB SCAV-3 (Li et al., 2016) (Fig. S2), as did control secreted mCherry fusions, consistent with the known acid-tolerance of the mCherry fluorophore (Clancy et al., 2023). These data are consistent with both LPR-1 and LPR-3 trafficking to the lysosome at the end of the molt cycle, as is typical of many pre-cuticle proteins (Sundaram and Pujol, 2024).

### LPR-1 promotes LPR-3 matrix incorporation

**Fig. 2.**
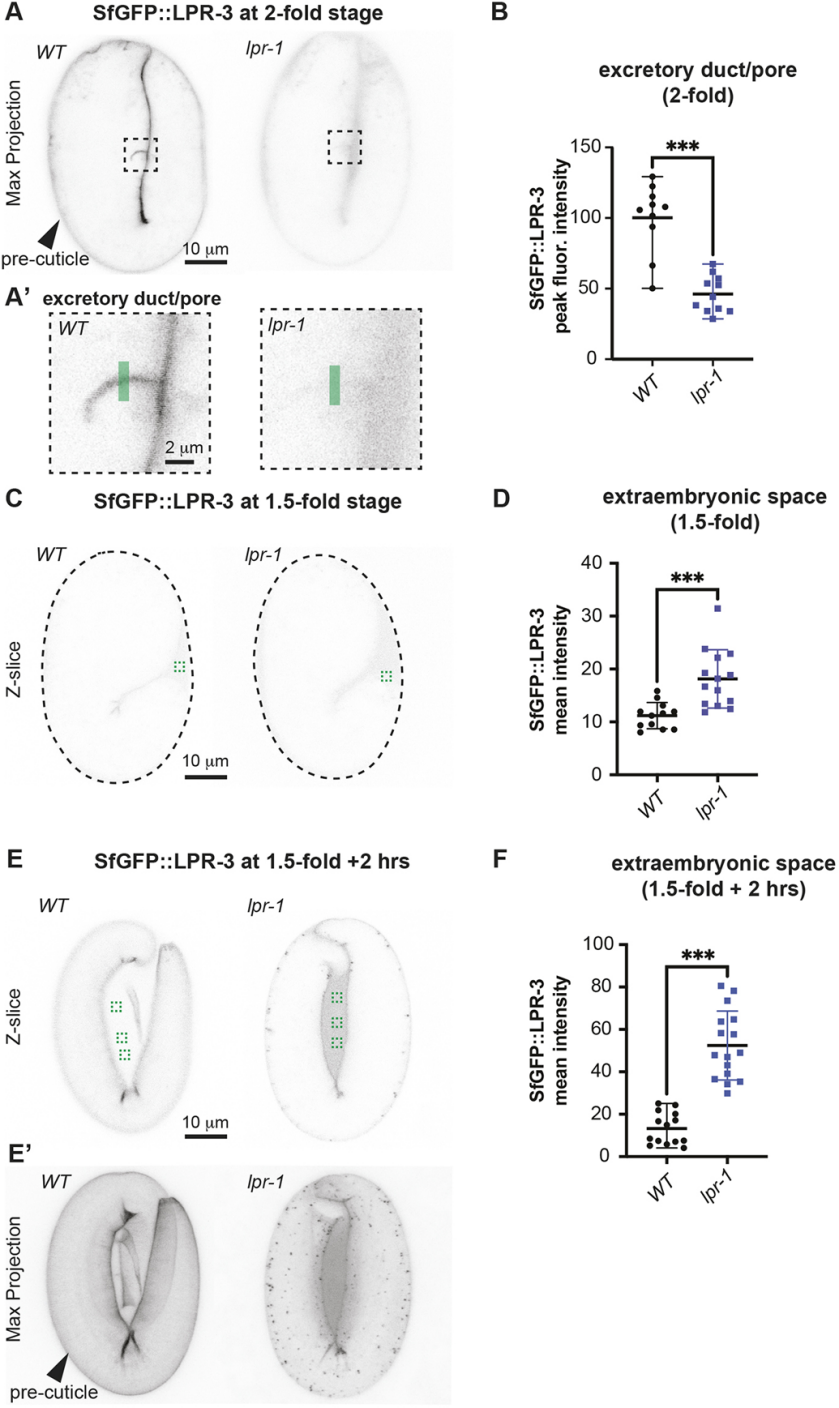
**LPR-1 promotes LPR-3 localization to the pre-cuticle matrix.** (A,B) Loss of *lpr-1* reduces LPR-3 accumulation within the developing duct and pore tubes. (A) Inverted projections of five confocal *z*-slices from 2-fold embryos. Boxed region is shown magnified in A′. Green line indicates duct/pore lumen region analyzed in B. (B) Peak intensities of SfGFP::LPR-3 duct/pore signal as assessed using the Plot Profile tool in FIJI. ***P<0.0001, Mann–Whitney *U*-test. (C-F) *lpr-1* mutants have elevated levels of LPR-3 accumulation within the extra-embryonic space, suggesting a post-secretory defect in matrix assembly. (C,E) Inverted single confocal *z*-slices through the medial region of 1.5-fold (C) and 1.5-fold+2 h (E) embryos. Dashed lines outline the embryos in C, as LPR-3 signal is faint at this stage. Green box(es) indicate regions analyzed in D,F. (E′) Maximum projections of the same embryos shown in E. The epidermal pre-cuticle signal also appears disorganized in *lpr-1* mutants compared to WT, and the presence of abundant puncta suggests premature LPR-3 endocytosis. (D,F) Mean intensities of SfGFP::LPR-3 extra-embryonic signal as assessed using the Measure mean gray value tool in FIJI. ***P=0.0001, Mann–Whitney *U*-test. Data are mean±s.d. All images are representative of at least *n*=10 images per genotype.

Unlike LPR-3, LPR-1 did not detectably incorporate into the matrix at any stage examined (Fig. 1D-G; Fig. S1). Nevertheless, *lpr-1* mutant embryos had less overall accumulation of LPR-3 within the lumens of the developing excretory duct and pore tubes (Fig. 2A,B) and in the aECMs of other tissues such as the epidermis (Fig. 2E′; see below). *lpr-1* mutants had progressively increased levels of extra-embryonic (non-matrix) LPR-3 (Fig. 2C-F) and more apical puncta in the epidermal cytoplasm (Fig. 2E′), indicating that secreted LPR-3 was not efficiently joining the pre-cuticle and that potentially it was being prematurely endocytosed and cleared. We conclude that LPR-1 plays an accessory role in pre-cuticle assembly, functioning upstream of LPR-3. Because *lpr-3* mutants also die due to excretory duct collapse amongst other problems (Forman-Rubinsky et al., 2017), defects in LPR-3 matrix assembly within the excretory duct and pore can explain the lethal phenotype of most *lpr-1* mutants.

### Loss of the scavenger receptor SCAV-2 bypasses the requirement for LPR-1 and restores LPR-3 to the excretory duct/pore matrix

**Fig. 3.**
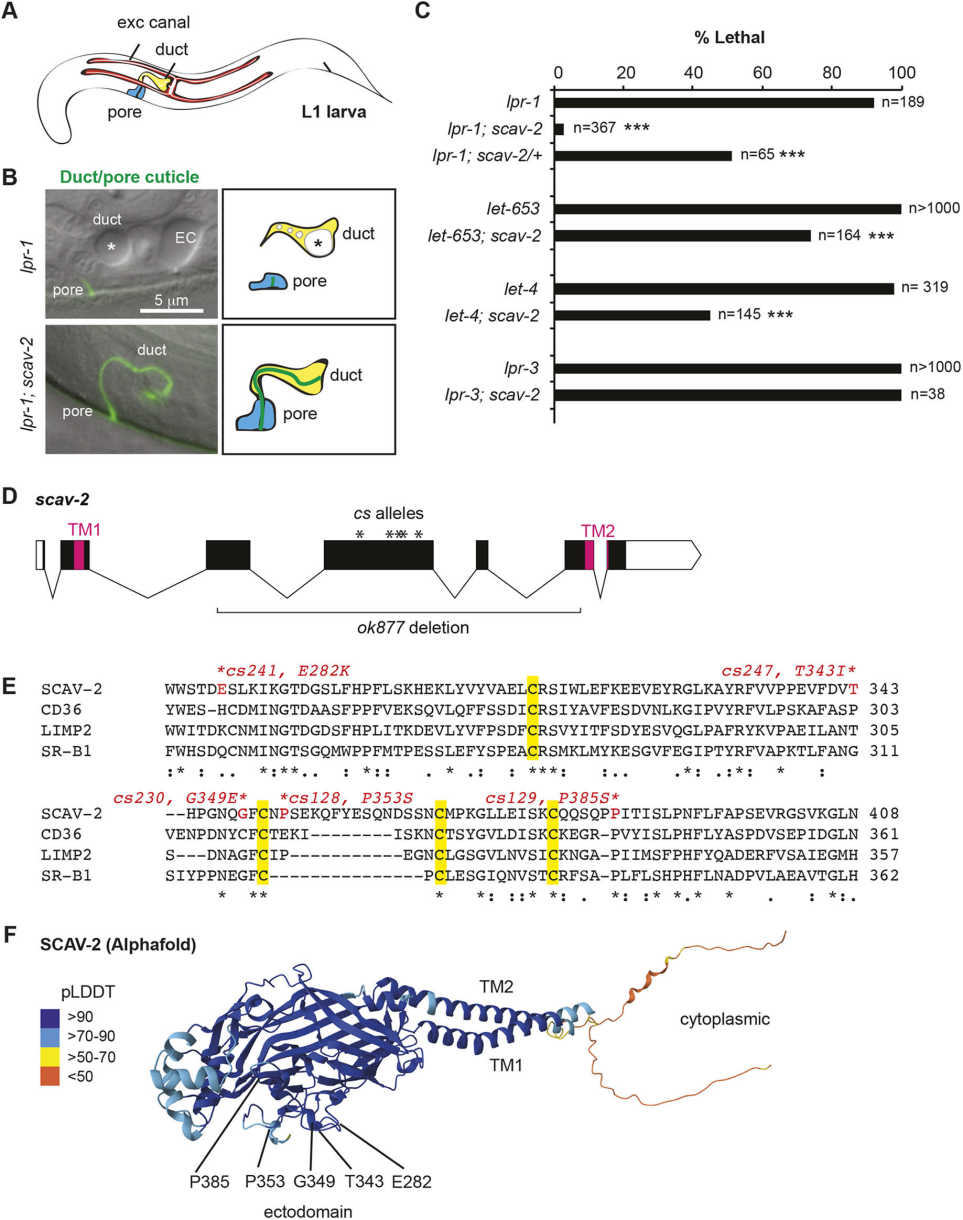
**Loss of *scav-2* suppresses the lethal excretory tube defects of *lpr-1* and some pre-cuticle mutants.** (A) Cartoon of L1 larva, showing unicellular tubes of the excretory system. The excretory duct and pore tubes are lined by cuticle, while the upstream excretory canal cell has a different type of aECM (Sundaram and Buechner, 2016). (B) Epifluorescence and differential interference contrast (DIC) merged images of L1 larvae bearing an SfGFP::GRL-2 marker of the duct and pore cuticle (Serra et al., 2024), with accompanying cartoons of the cells. *lpr-1* mutants lose duct and pore tube lumen integrity (Pu et al., 2017; Stone et al., 2009) and have a large dilation but little or no cuticle in the remaining duct lumen (asterisk). Dilations are also present within the excretory canal lumen (EC). Loss of *scav-2* restored a continuous duct and pore lumen and cuticle (*n*=11/11), as in WT larvae. (C) *scav-2(ok877)* partially suppressed the excretory lethal phenotype of *lpr-1(cs207), let-653(cs178)* and *let-4(mn105)* mutants, but not *lpr-3(cs144)* mutants. ***P<0.0001, Fisher's exact test, compared to *scav-2(+)* control. (D) Diagram of *scav-2* locus, showing locations of molecular lesions and regions encoding both transmembrane (TM) domains. See also Fig. S3. (E) Partial alignment of the SCAV-2, CD36, LIMP2 and SR-B1 ectodomains, showing conserved cysteines (yellow) and locations of *scav-2* missense lesions (red). This entire region is deleted by *ok877* (see Fig. S3). Alignment generated with Clustal Omega (Sievers and Higgins, 2021). (F) Alphafold predicted structure of SCAV-2 (Senior et al., 2020), showing positions of the *scav-2* missense alleles. The structure is colored according to the Alphafold pLDDT score, a measure of prediction confidence, which in this case is very high for most regions.

To better understand how LPR-1 affects the pre-cuticle matrix, we conducted an ethyl methanesulfonate (EMS) mutagenesis screen to find suppressors of *lpr-1* mutant lethality. Approximately 90% of *lpr-1* mutants die as first stage (L1) larvae due to the aforementioned discontinuities in the excretory duct and pore lumens, whereas the remaining 10% maintain tube patency and survive to adulthood (Fig. 3A-C) (Pu et al., 2017; Stone et al., 2009). After screening ∼10,000 mutagenized genomes, we identified nine suppressors that increased *lpr-1* survival to >50% (see Materials and Methods; Fig. S3). Four of the best suppressors contained independent missense changes in the gene *scav-2*, and at least two of these were semi-dominant suppressors (Fig. 3D,E; Fig. S3). An independent *scav-2* deletion allele, *ok877*, was also a semi-dominant suppressor of *lpr-1* lethality (Fig. 3C) and restored normal duct and pore lumen morphology to *lpr-1* null mutants (Fig. 3B). These data confirm that loss of *scav-2* activity is responsible for the suppressor phenotype and demonstrate that *scav-2* is haplo-insufficient and therefore dose-sensitive for that phenotype. *scav-2(ok877)* mutants did not show any obvious developmental phenotype in an *lpr-1(+)* background.

*scav-2* encodes a scavenger receptor of the SCARB family, with two predicted transmembrane domains flanking a central ectodomain, and short N- and C-terminal cytoplasmic tails (Fig. 3D,F). At the amino acid level, SCAV-2 is equally similar to mammalian CD36 and LIMP2 (also known as Scarb2) (∼28% identical) and slightly less similar to SR-B1 (Scarb1) (∼24% identical). These three mammalian SCARBs can each bind specific lipids or lipoproteins and facilitate their transport into or across cellular membranes (Glatz et al., 2022; Heybrock et al., 2019; Linton et al., 2017; Reczek et al., 2007). The central ectodomain of these receptors forms a hydrophobic tunnel structure through which lipids may access the membrane (Hsieh et al., 2016; Neculai et al., 2013). Interestingly, most of the *scav-2* missense alleles disrupt ectodomain residues that are identical between SCAV-2 and LIMP2 (Fig. 3E) and all cluster along one external surface of the predicted ectodomain structure (Fig. 3F), suggesting this region may be particularly important for interaction with a relevant partner or cargo. These missense alleles may be either loss of function or dominant negative; they have not been further characterized. *scav-2(ok877)* deletes most of the ectodomain and leads to a frameshift and premature stop (Fig. 3D), likely leading to nonsense-mediated decay; all further experiments were performed using this putative null allele.

**Fig. 4.**
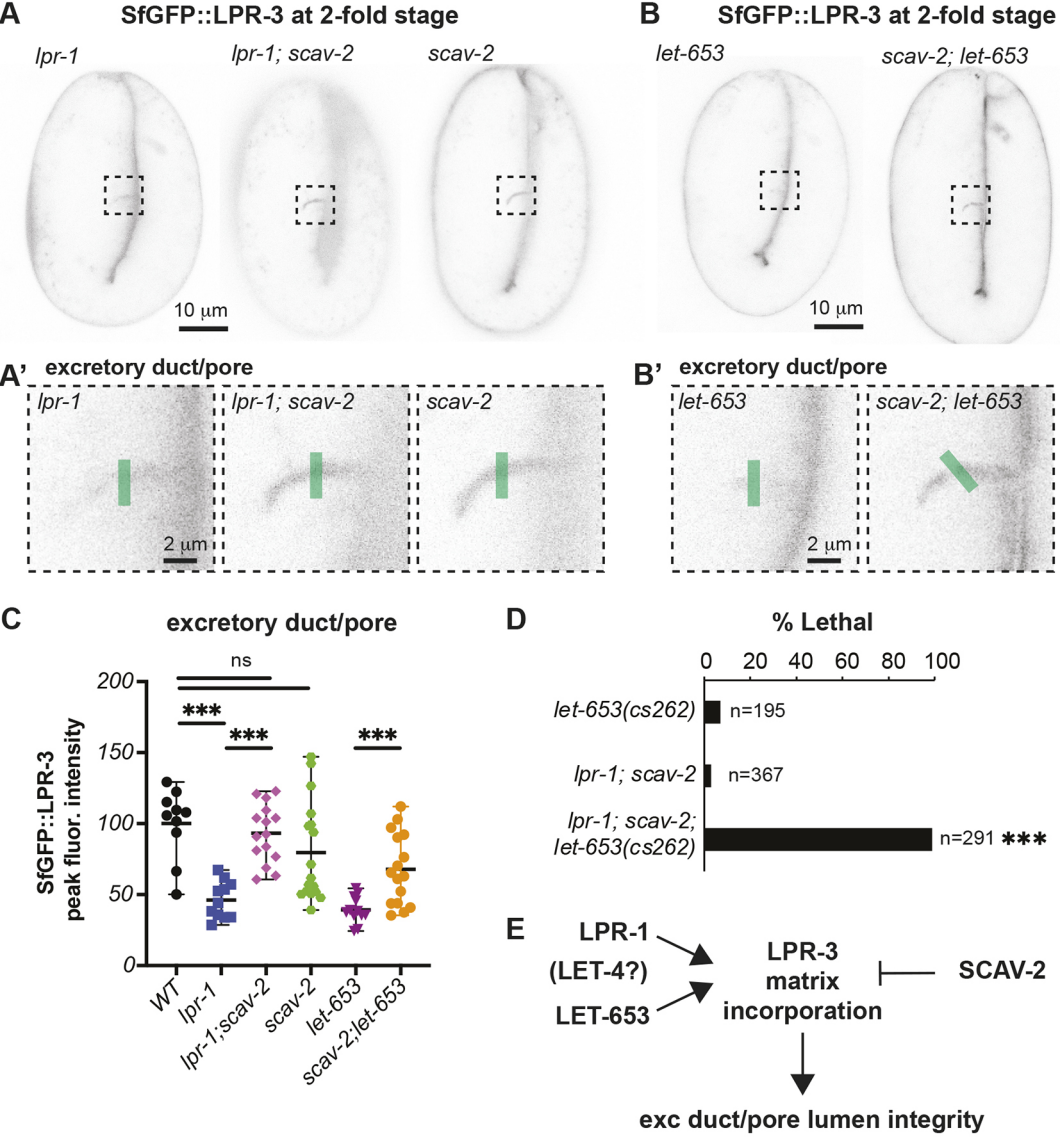
**Loss of *scav-2* restores LPR-3 to the excretory duct/pore matrix of *lpr-1* and *let-653* mutants.** (A,B) Loss of *scav-2* restores LPR-3 accumulation within the developing duct and pore tubes. Inverted projections of five confocal *z*-slices from 2-fold embryos. Boxed regions are shown magnified in A′,B′, respectively. Green lines indicate duct/pore lumen region analyzed in C. (C) Peak intensities of SfGFP::LPR-3 duct/pore signal as assessed using the Plot Profile tool in FIJI. ***P<0.0001, Mann–Whitney *U*-test. Control data are reproduced from Fig. 2B for comparison. Data are mean±s.d. (D) Loss of *scav-2* cannot suppress *lpr-1* lethality when *let-653* function is also partly compromised by *let-653(cs262 [LET-653::SfGFP]).* (E) Genetic model of relationships.

While loss of *lpr-1* disrupted LPR-3 matrix localization (Fig. 2), loss of *scav-2* restored LPR-3 to the excretory/duct lumen in *lpr-1* mutants (Fig. 4A,C). Loss of *scav-2* did not increase LPR-3 levels in an otherwise wild-type (WT) background (Fig. 4A,C), suggesting either that LPR-3 was already at its maximal level or that SCAV-2 does not directly transport LPR-3 but instead affects an LPR-3 partner (see Discussion). Irrespective of the specific SCAV-2 cargo, these results indicate that LPR-1 and SCAV-2 function in opposition to respectively promote and inhibit LPR-3 matrix localization and the protection of narrow tube integrity.

### Loss of SCAV-2 suppresses lethality of *let-653* and *let-4* pre-cuticle mutants, but not *lpr-3* mutants

In addition to LPR-3, the ZP domain protein LET-653 and the eLRRon protein LET-4 are pre-cuticle components that are essential for excretory duct and pore lumen integrity (Gill et al., 2016; Mancuso et al., 2012). We identified a fifth *scav-2* allele, *cs230*, in an independent EMS-mutagenesis screen for suppressors of *let-653* lethality (Fig. 3D,E; Fig. S3; see Materials and Methods). The *scav-2*(*ok877)* null allele also partly suppressed lethality of *let-653* and *let-4* null mutants, though not of *lpr-3* mutants (Fig. 3C). Like *lpr-1* mutants, *let-653* mutants had greatly reduced LPR-3 in the excretory duct/pore lumen, but loss of *scav-2* partly restored LPR-3 to the lumen (Fig. 4B,C). Therefore, loss of *scav-2* ameliorates the requirements for multiple factors that act upstream of LPR-3.

Loss of LPR-1 had little or no effect on the duct/pore localization of either LET-653 or LET-4 fusions (Fig. S4), suggesting that LPR-1 acts at a step downstream or in parallel to these other matrix factors. Notably, we used a transgenic reporter for the LET-653 studies, because we could not recover any viable *lpr-1* mutants expressing an endogenously tagged LET-653::SfGFP fusion. Furthermore, most *lpr-1; scav-2*; LET-653::SfGFP larvae also died with excretory tube defects, despite the fact that most *lpr-1; scav-2* double mutants and most control LET-653::SfGFP animals are viable and healthy (Fig. 4D). This synthetic lethal interaction indicates that *lpr-1* and *let-653* act in parallel pathways and that *lpr-1* mutants are highly sensitive to even minor perturbations to LET-653 function introduced by the tag; loss of *scav-2* can compensate for loss of either the LPR-1 or LET-653 pathways, but not for simultaneous perturbations in both. We conclude that LPR-1 and LET-653 (and perhaps also LET-4) cooperate to facilitate LPR-3 assembly in the duct/pore aECM, while SCAV-2 opposes their ability to do so (Fig. 4E).

### Loss of SCAV-2 does not globally restore matrix structure to *lpr-1* mutants

**Fig. 5.**
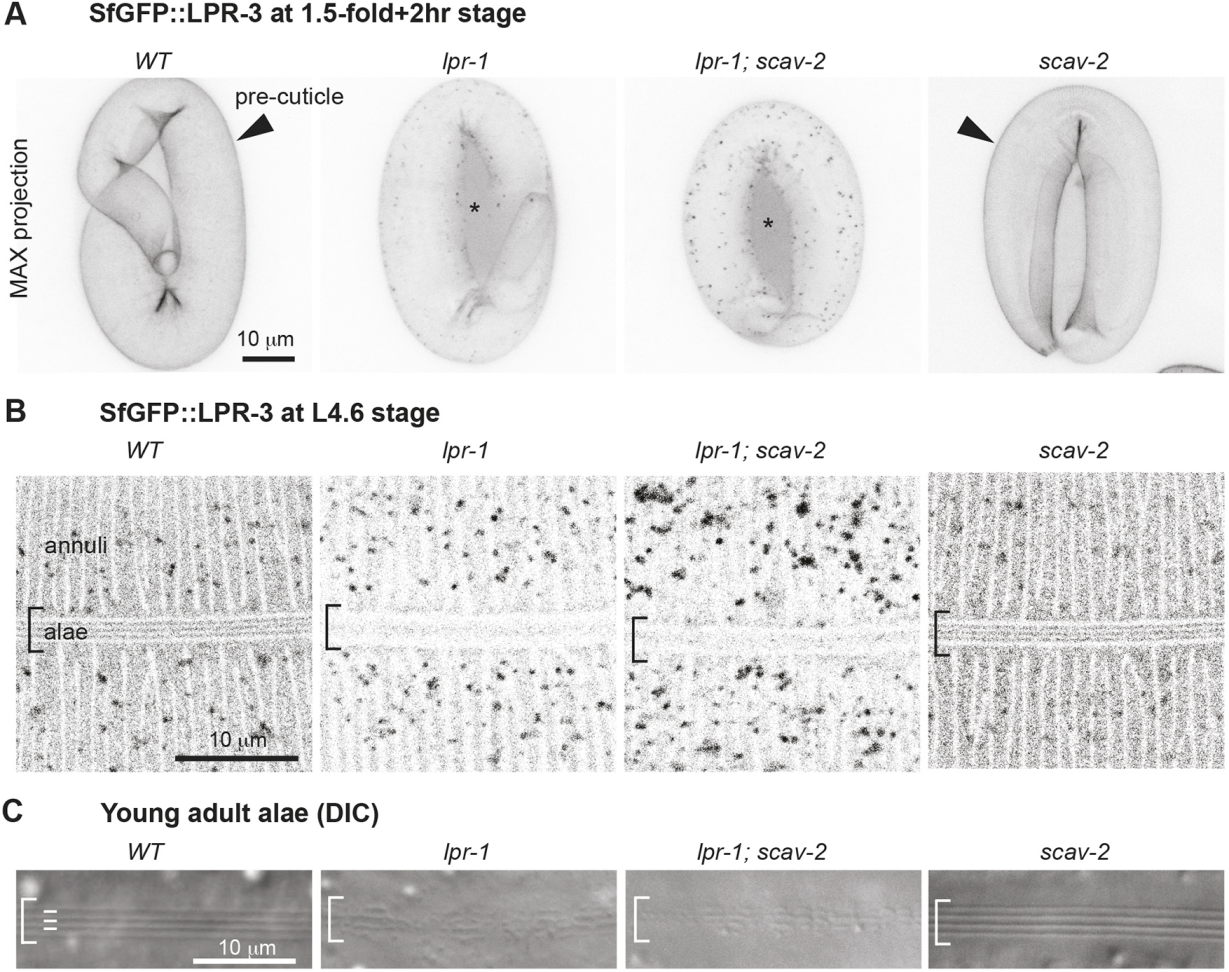
**Loss of *scav-2* does not suppress *lpr-1* epidermal matrix defects.** (A) Inverted confocal maximum projections of SfGFP::LPR-3 in the embryonic pre-cuticle (arrowhead). *lpr-1* and *lpr-1; scav-2* mutants have reduced SfGFP::LPR-3 matrix signal and increased puncta and extra-embryonic signal (asterisk). (B) Inverted confocal *z*-slices of SfGFP::LPR-3 in the epidermal pre-cuticle of L4.6 stage larvae. *lpr-1* and *lpr-1; scav-2* animals have reduced SfGFP::LPR-3 matrix signal in circumferential annuli and complete loss of the organized longitudinal stripe pattern in the developing alae (brackets). (C) DIC images of the alae in young adults. *scav-2* does not suppress the *lpr-1* alae defect. All images are representative of at least *n*=10 per genotype.

In addition to affecting the excretory duct and pore matrix, *lpr-1* mutants also showed reduced and disorganized patterns of SfGFP::LPR-3 localization in epidermal pre-cuticles in both embryos and L4 larvae (Fig. 2E′, Fig. 5A,B). SfGFP::LPR-3 still labeled circumferential pre-cuticle annuli (but not the intervening furrows) over the main hypodermis (see Fig. 1E) as in wild type, but the annular signal appeared to be weaker, while various intracellular puncta were present prematurely (Fig. 5A,B). SfGFP::LPR-3 disorganization was most severe over the lateral (seam) epidermis at the L4 stage (Fig. 5B), with a complete loss of the longitudinal stripe pattern, consistent with previous reports that *lpr-1* mutants also have structural defects in the lateral alae ridges of the adult cuticle (Forman-Rubinsky et al., 2017; Katz et al., 2022). Loss of *scav-2* did not suppress the aberrant epidermal pattern of SfGFP::LPR-3 (Fig. 5A,B) nor the cuticle alae defects (Fig. 5C) of *lpr-1* mutants. Therefore, loss of *scav-2* does not globally restore aECM structure, but instead specifically compensates for defects in aECM-dependent protection of the narrow excretory duct and pore tubes.

### *lpr-1* and *scav-2* mutants do not have global metabolic changes

Since lipocalins and SCARBs are best known as lipid transporters, we compared WT, *lpr-1* mutants and *lpr-1; scav-2* double mutants by lipidomic profiling using liquid chromatography and mass spectrometry (LC-MS). We focused on 1.5-fold embryos for these experiments, since previous genetic rescue data indicated that the LPR-1 excretory tube-shaping function is required zygotically at or before this stage (Stone et al., 2009). These experiments did not detect any consistent differences in lipid content among genotypes (Fig. S5). Furthermore, dietary supplementation assays failed to detect any significant effect of manipulating cholesterol or the major phospholipids phosphatidylcholine (PC) or phosphatidylethanolamine (PE) on the *lpr-1* mutant phenotype (Fig. S6A,B,E). Genetic mutation of *pcyt-1* (encoding phosphocholine cytidylyltransferase) also had no effect (Fig. S6C), while loss of *pld-1* (encoding phospholipase D) only slightly improved *lpr-1* survival (Fig. S6D). We previously reported that *lpr-1* mutants have normal epidermal barrier function (Forman-Rubinsky et al., 2017), which depends on a lipid-rich outer cuticle layer (Bada Juarez et al., 2019; Blaxter, 1993; Njume et al., 2022). While they do not exclude effects on low abundance lipids or on lipid localization, as opposed to abundance (as might be predicted for lipid transporters), these experiments suggest that *lpr-1* and *scav-2* mutants do not have broad metabolic dysregulation.

### SCAV-2 acts cell autonomously in the duct and pore tubes

**Fig. 6.**
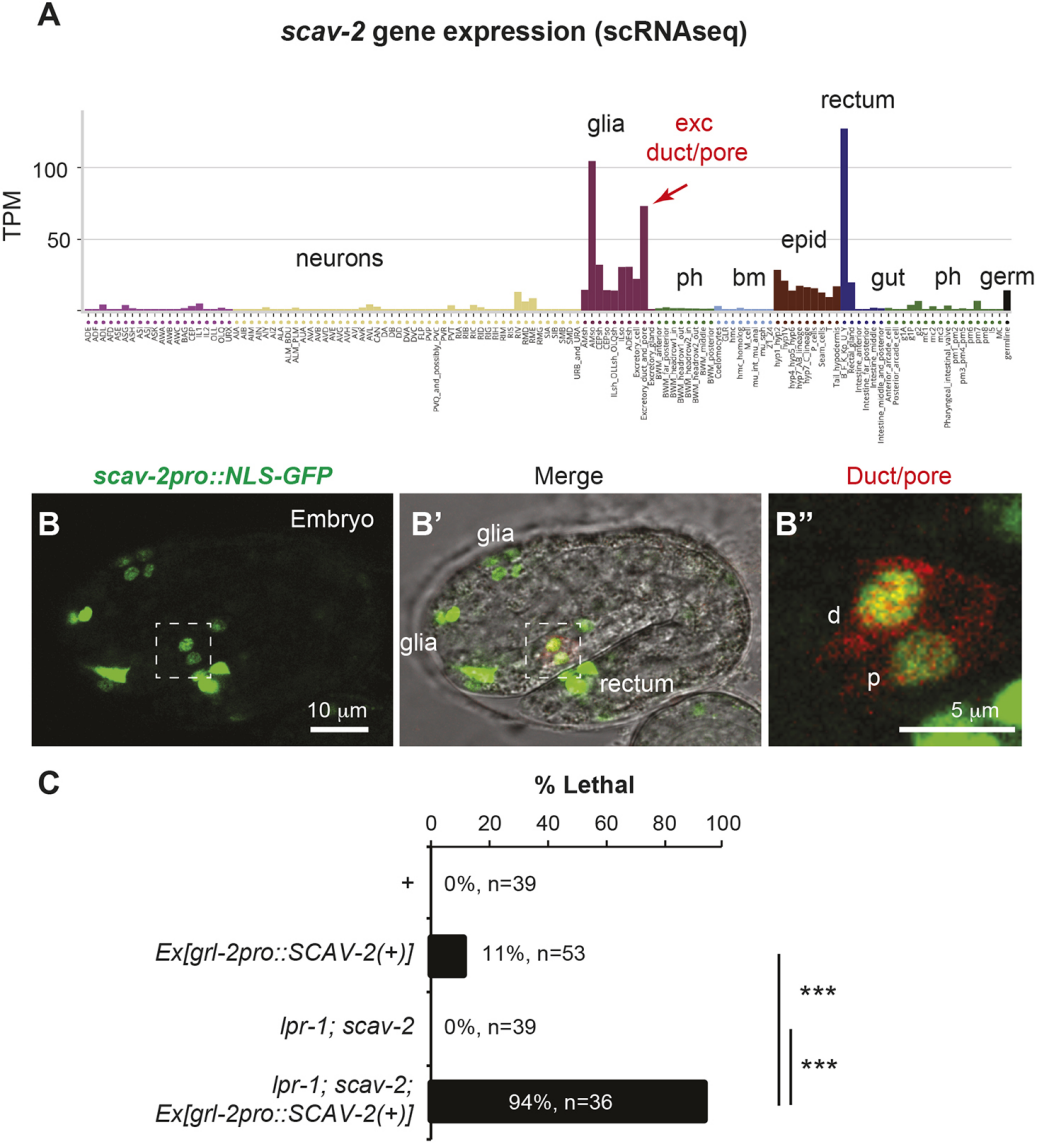
***scav-2* is expressed and functions locally within the excretory duct and pore tubes.** (A) The excretory duct and pore are among the cells with highest levels of *scav-2* expression. Summary of *scav-2* expression (transcripts per million) in embryonic cell types, based on scRNAseq data from Packer et al. (2019) and Large et al. (2025). Plot generated with tool from Large et al. (2025). (B) A *scav-2pro::NLS-GFP* transcriptional reporter (green) is expressed in the duct (d) and pore (p), marked by *grl-2pro::mRFP* (red) in B′,B″. Image is representative of at least *n*=10 embryos examined. (C) A *grl-2pro::SCAV-2(+)* transgene caused minimal lethality in a WT background, but rescued the *scav-2* suppressor phenotype, restoring excretory defects and lethality to an *lpr-1; scav-2* strain. Transgenic and non-transgenic siblings were scored in parallel. ***n<0.001, Fisher's exact test.

To ask in which cells SCAV-2 is expressed and where it is relevant, we analyzed available single cell RNA sequence (scRNAseq) data from embryos (Packer et al., 2019), generated a transcriptional reporter and conducted tissue-specific rescue experiments. Both the scRNAseq data and the transcriptional reporter indicated that, in embryos, *scav-2* is expressed most highly in interfacial tubes, including the rectum, glial sheath and socket cells, and in the excretory duct and pore cells beginning early in tube development (Fig. 6A,B). Some expression was also detected in the epidermis. A *grl-2* promoter transgene driving expression of the *scav-2* cDNA only in the excretory duct and pore and several sensory glia (Fung et al., 2020) restored excretory failure and lethality to *lpr-1; scav-2* double mutants, indicating rescue of the *scav-2* suppressor phenotype and consequent reversion to an *lpr-1* mutant phenotype (Fig. 6C). This transgene caused very little lethality in a WT background (Fig. 6C), indicating that it is not generally toxic. We conclude that *scav-2* can function cell autonomously in the excretory duct and pore to oppose LPR-1 and aECM factors.

### SCAV-2 localizes to apical plasma membranes

**Fig. 7.**
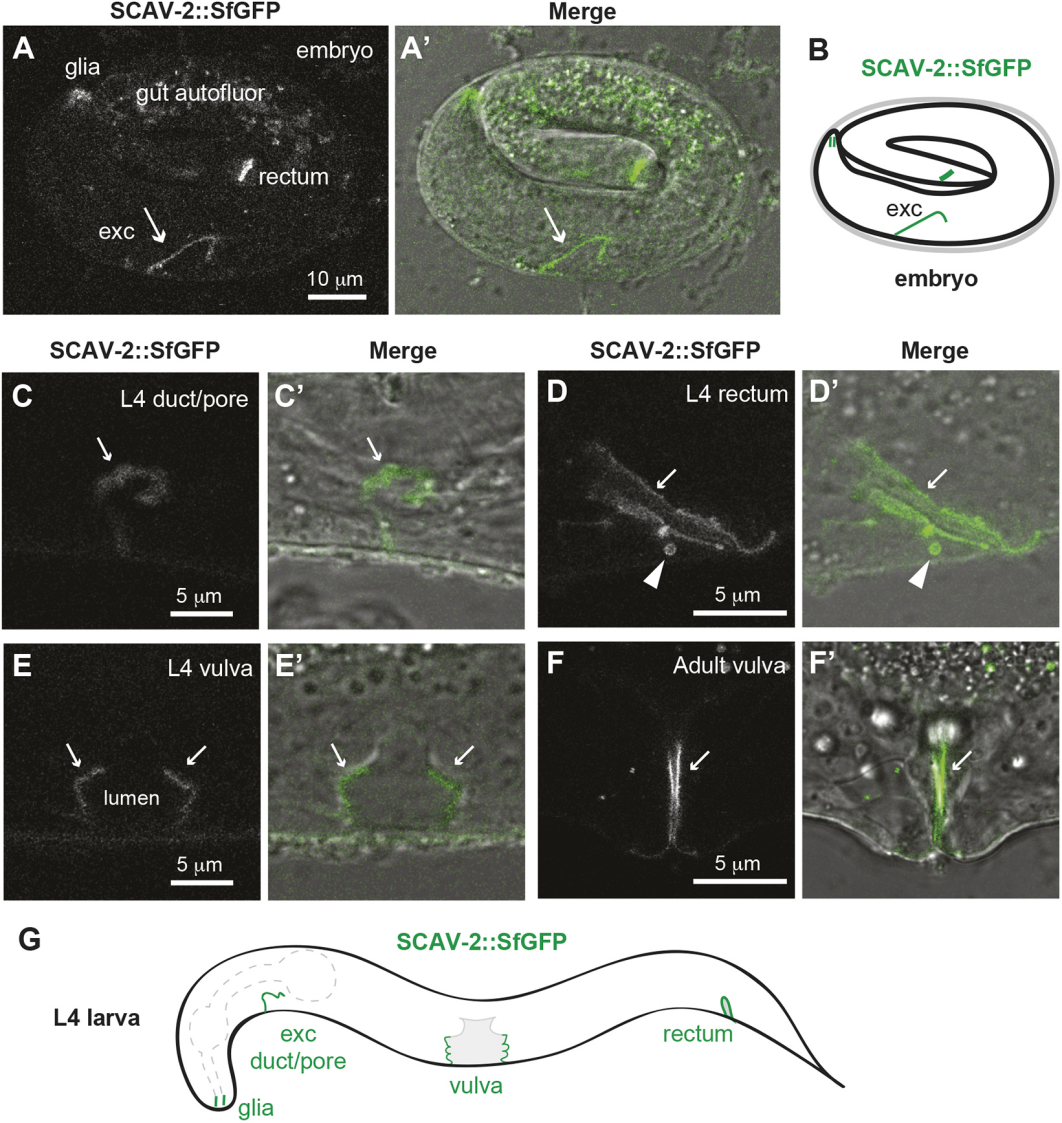
**SCAV-2 localizes to apical membranes of interfacial tubes.** (A,A′) 3-fold embryo showing SCAV-2::SfGFP marking the apical domain of the excretory duct and pore tubes (arrow) along with the rectum and glia. (B) Schematic summary of observed SCAV-2::SfGFP expression in embryos. (C-F′) L4 larvae showing SCAV-2::SfGFP marking the apical domains of the duct and pore (C,C′), rectum (D,D′) and vulva (E,E′) tubes. (F,F′) SCAV-2::SfGFP persists in the adult vulva. Arrows indicate apical membrane signals and the arrowhead in D indicates a large intracellular vesicle. All images are representative of at least *n*=10 specimens imaged per stage and tissue. (G) Schematic summary of observed SCAV-2::SfGFP expression at the L4 stage.

SCARB proteins have been observed on both plasma membranes and internal organelle membranes, and are therefore hypothesized to transport cargo between various different compartments (Glatz et al., 2022; Gomez-Diaz et al., 2016; Heybrock et al., 2019; Li et al., 2016; Linton et al., 2017; Neculai et al., 2013; Pinheiro et al., 2023; Wingen et al., 2017). To assess where SCAV-2 localizes within cells *in vivo*, we used CRISPR-Cas9 to insert a fluorescent SfGFP tag at the C-terminus within the endogenous locus. The SCAV-2::SfGFP knock-in was functional based on its failure to suppress *lpr-1* defects {92% of *lpr-1; scav-2(cs256 [SCAV-2::SfGFP])* animals arrested as larvae, *n*=166}. In both WT and *lpr-1* mutant backgrounds, SCAV-2::SfGFP localized to the apical membranes of the excretory duct and pore (Fig. 7A-C) and other interfacial tubes such as the rectum and vulva (Fig. 7D-G). It was observed only rarely on internal vesicles (e.g. Fig. 7D), which may be involved in its delivery to or removal from the plasma membrane. Unlike transient pre-cuticle factors, SCAV-2 apical localization persisted throughout the molt cycle and continued into adulthood, when it became especially prominent in the vulva (Fig. 7F). We conclude that SCAV-2 is most appropriately positioned to transport materials between the aECM region and the cell interior.

## DISCUSSION

**Fig. 8.**
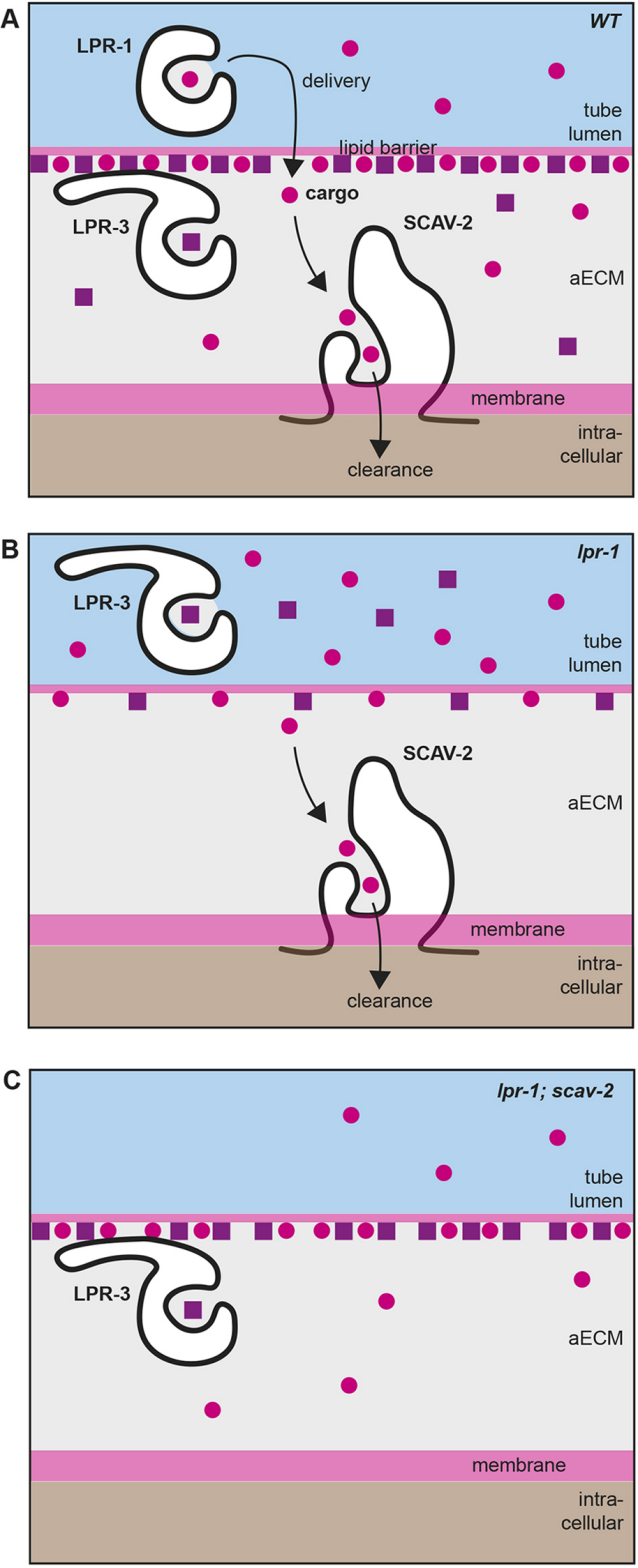
**Model for relationships among LPR-1, LPR-3 and SCAV-2.** (A) LPR-1 and SCAV-2 function to deliver and clear, respectively, an unknown lipophilic cargo (pink circles) that helps to promote assembly or retention of LPR-3 in the aECM. It is also possible that LPR-1 and SCAV-2 transport distinct cargos that each impact LPR-3 (not shown). LPR-3 matrix association is conferred by its unique N-terminal domain (Forman-Rubinsky et al., 2017). LPR-3 is presumed to carry a different cargo (purple squares) since the LPR-1 and LPR-3 cup domains are not interchangeable (Forman-Rubinsky et al., 2017). (B) In the absence of LPR-1, cargo levels drop below the level needed to efficiently recruit or retain LPR-3 in the matrix. We infer that some amount of this cargo must still reach the aECM independently of LPR-1, but it is then actively removed by SCAV-2, keeping its levels low. (C) In the absence of both LPR-1 and SCAV-2, cargo levels are restored to homeostasis and LPR-3 can assemble in the aECM.

aECMs play important roles in shaping and protecting tube lumens and are particularly crucial for maintaining patency and flow in the narrowest tubes such as capillaries and alveoli (Butler et al., 2020; Gill et al., 2016; Henderson-Toth et al., 2012; Jazwinska et al., 2003; Shi et al., 2025; Whitsett et al., 2015). This work shows that two putative lipid or lipoprotein transporters, the lipocalin LPR-1 and the CD36-related scavenger receptor SCAV-2, function in opposition to affect aECM assembly and aECM-dependent narrow tube patency in *C. elegans*. The relevant cargos of these transporters are unknown but may include LPR-3 or other cofactors or lipids that contribute to LPR-3 matrix assembly. LPR-1 normally promotes the delivery of such factors to increase the LPR-3 content and tube-widening properties of aECM, while SCAV-2 normally removes such tube-widening factors from the luminal environment (Fig. 8). If mammalian lipocalins and SCARBs similarly affect aECM organization in the microvasculature and other tubular organ systems, such a role could explain many of their reported impacts on human health.

### Lipocalin LPR-1 promotes LPR-3 matrix incorporation

In *lpr-1* mutants, LPR-3 is still apically secreted but does not efficiently incorporate into the pre-cuticle aECM, both in the excretory duct/pore and in other parts of the body. Therefore, LPR-1 must act upstream of LPR-3 to promote its matrix delivery or incorporation (Fig. 8). LPR-1 is only one of multiple factors with this role, since its loss reduces but does not eliminate LPR-3 matrix incorporation. Indeed, LPR-1 acts in parallel to the ZP domain protein LET-653, a component of the pre-cuticle that also promotes LPR-3 matrix incorporation (Fig. 4), presumably by organizing an appropriate matrix environment.

The low abundance and non-cell autonomous action of LPR-1 are most consistent with a non-structural matrix role such as in long-distance transport or signaling (Pu et al., 2017; Stone et al., 2009). Lipocalins can function as carriers of many different types of hydrophobic cargoes, including steroid hormones or other signaling lipids (Yang et al., 2023). For example, mammalian ApoM transports sphingosine-1-phosphate (S1P) for signaling via the S1P receptor (Christensen et al., 2016; Christoffersen et al., 2011), while retinol binding protein (RBP4) transports retinol through the bloodstream and facilitates its uptake through receptor STRA6 for eventual signaling through the retinoic acid receptor (Kawaguchi et al., 2007; Steinhoff et al., 2021). Although some evidence suggests that *C. elegans* LPR-1 and LPR-3 can also bind and transport S1P, mutations in the S1P pathway do not cause the lethal excretory duct phenotypes seen in *lpr-1* and *lpr-3* mutants (Wu et al., 2025). Several essential *C. elegans* nuclear hormone receptors (NHRs) do promote matrix-relevant gene expression during the molt cycle (Antebi, 2006; Johnson et al., 2023), so LPR-1 could potentially transport a relevant NHR ligand; however, no such ligand has been identified to date and our cholesterol manipulation experiments (Fig. S6E) did not support sterol involvement. An alternative model is that LPR-1 more directly delivers specific matrix lipids or lipoproteins, such as LPR-3 or an LPR-3 cofactor, that are required within the luminal environment of the excretory duct and pore tubes (Fig. 8). Studies of the other *lpr-1* suppressors (Fig. S3A) may provide further clues to the LPR-1 cargo.

The frequent association of both LPR-1 and LPR-3 with epidermal lysosomes or LROs may be significant. In addition to being sites for protein degradation, lysosomes are important sites of lipid storage and metabolism (Thelen and Zoncu, 2017). Many epithelia also have specialized LROs dedicated to their particular needs; for example, lamellar bodies in the lung are specialized LROs that store, modify, and deliver lipid-rich lung surfactant (Sever et al., 2021), and *C. elegans* gut granule LROs are known sites of BODIPY-labeled lipid accumulation and ascaroside (glycolipid) biosynthesis (Hermann et al., 2005; Le et al., 2020; Panda et al., 2017). *C. elegans* epidermal lysosomes or LROs are diverse in size, morphology and spatial distribution (Fig. S2) and have been noted to change appearance over the course of the molt cycle (Miao et al., 2020). An attractive hypothesis is that LPR-1 could be involved in transporting materials to or from a subset of these lysosomes/LROs that store and recycle aECM factors. Further studies of these compartments will be important to understand their potential functions in matrix handling.

### SCARB SCAV-2 opposes LPR-1 by promoting LPR-3 matrix removal

Scavenger receptors are so named based on their ability to remove various factors from the extracellular environment (PrabhuDas et al., 2017). The data presented here suggest that *C. elegans* SCAV-2 acts locally to remove factors from the luminal matrix in the excretory duct and pore (Fig. 8A,B). The SCAV-2 cargo could be either the same or distinct from the LPR-1 cargo, but it must be present within the luminal aECM to some degree, even in the absence of LPR-1 (Fig. 8B), so that it can accumulate in the absence of both transporters (Fig. 8C). Loss of *scav-2* suppresses *lpr-1* tube defects and restores LPR-3 to the luminal matrix (Fig. 8C), implicating LPR-3 as a possible SCAV-2 cargo. However, we do not have evidence to favor this model, as LPR-3 matrix levels were not increased in *scav-2* single mutants; instead SCAV-2 may remove matrix lipids or other factors that function upstream to impact LPR-3 recruitment or retention (Fig. 8).

SCARB proteins are best known for their lipid and lipoprotein transport roles but could have broader functions in matrix biology. The mammalian SCARBs CD36 and SR-B1 are found on plasma membranes and are important for cellular uptake of circulating fatty acids and LDL or HDL cholesterol (Febbraio et al., 1999; Rigotti et al., 1997; Varban et al., 1998). However, they can also bind other types of molecules, including aECM protein cargoes such as thrombospondin or collagen (Asch et al., 1993). The SCARB LIMP2 localizes to endomembranes and functions both as a cholesterol transporter and as a sorting receptor that traffics specific lipid-modifying enzymes to lysosomes and LROs (Heybrock et al., 2019; Reczek et al., 2007); because LROs include surfactant-producing lamellar bodies, LIMP2 can moderate the lipid content of lung surfactant (Kook et al., 2016). A *Drosophila* SCARB, Emp, was recently shown to promote clearance of LDLr domain-containing proteins during maturation of the aECM within tracheal airway tubes (Pinheiro et al., 2023). Thus, there may be very broadly conserved roles for SCARBs in modulating aECM organization.

### Potential aECM-related roles for lipocalins and SCARBs in human disease

As mentioned in the Introduction, there are many reported correlations or anti-correlations between lipocalin or SCARB levels and tissue damage and susceptibility to infection or disease in patient populations, as well as in animal models (Glatz et al., 2024; Koenig et al., 2021; Linton et al., 2017; Paragas et al., 2012). For example, for various lipocalins, either reduced or increased levels of expression have been noted to correlate with cardiovascular disease burden and, in some cases, contribute to it (Yang et al., 2023), while reducing CD36 levels can either predispose or protect from cardiovascular disease depending on genetic background and diet (Glatz et al., 2024). A wide range of mechanisms have been proposed to explain these observations, most involving effects on bacterial siderophores, inflammatory signaling pathways, or lipid metabolism and storage (Glatz et al., 2024; Yang et al., 2023). Mammalian studies rarely examine the vascular aECM, which is difficult to visualize by light microscopy, but disruption or repair of that aECM could easily contribute to many of the other observed effects. The results here clearly demonstrate that both lipocalin and SCARB mutations impact aECM contents and organization in *C. elegans* and should motivate further exploration of this possibility in other systems.

## MATERIALS AND METHODS

### Animal husbandry

All strains used in this study are listed in Table S1. Unless otherwise indicated, strains were grown under standard conditions on NGM plates at 20°C (Brenner, 1974). All experimental planning relied on data publicly available through Wormbase and the Alliance of Genome Resources (Sternberg et al., 2024).

### Isolation of *scav-2* alleles

Strain UP1321 [*lpr-1(cs73)*] was mutagenized with 50 mM EMS (Brenner, 1974), and P0 animals were placed on large nematode growth medium (NGM) plates. After 2 weeks, plates were screened for increased population density and individual animals were picked to establish stocks; only a single suppressed stock from each original P0 plate was kept. For each established stock, survival was quantified by collecting eggs from at least five animals and assessing survival to L4 stage 3 days later. We estimate that progeny from ∼5000 F1s were screened, and a total of 14 suppressed stocks were established, for which nine showed survival >50% and were further studied (Fig. S3). *lpr-1* suppressors were tested for linkage to chromosomes I or III by outcrossing with balancer *hT2[qIs48] (I;III)* and then assessing what proportion of F2 non-balancer animals (*lpr-1* mutants) carried the suppressor. Four of the nine strains, which turned out to contain *scav-2* alleles, showed 100% presence of the suppressor, indicating linkage to the balancer-covered region.

Separately, strain UP2846 [*let-653(cs178) jcIs1; csIs61; csEx358 (lpr-1pro::let-653+; unc-119::gfp)*] was mutagenized with 50 mM EMS and the F2 progeny screened for any surviving non-transgenic larvae. We estimate that progeny from ∼5000 F1s were screened and a single suppressed stock (with suppressor *cs230*) was identified.

The *scav-2 cs128*, *cs129* and *cs230* lesions were identified by whole genome sequencing of Nextera libraries on an Illumina sequencer, followed by bioinformatic analysis with Cloudmap (Minevich et al., 2012). The *scav-2 cs241* and *cs247* lesions were identified (and the other lesions confirmed) by Sanger sequencing of PCR-amplified genomic fragments.

### *scav-2* transgenic reporters and rescue constructs

All plasmids for transgenic experiments were based on standard vector backbone pPD49.26 (Addgene), with promoter sequences cloned into MCSI and the gene of interest cloned into MCSII. See Table S2 for details.

### *lpr-1* and *scav-2* endogenous reporter fusions

Fluorescent tags were inserted into the 3′ end of the endogenous *lpr-1* locus using CRISPR-Cas9 and the self-excising cassette method (Dickinson et al., 2015) (see Table S2). After marker excision, edits were confirmed by Sanger sequencing.

An SfGFP fluorescent tag was inserted into the 3′ end of the endogenous *scav-2* locus using CRISPR-Cas9 and the scarless editing protocol of Dokshin et al. (2018), modified by using two crRNAs simultaneously. Cas9-NLS protein was purchased from the QB3 MacroLab core at the University of California, Berkeley (CA, USA). Ultramers, tracr RNA and crRNAs (Table S2) were purchased from Integrated DNA Technologies (IDT). Edits in F2s were identified by visual screening for fluorescence and were verified by Sanger sequencing.

### Microscopy

Epifluorescent and differential interference contrast images of the L1 duct and pore cuticle and adult alae were captured using a Zeiss Axioskop and a Leica DFC360 FX camera. Confocal images were captured using a Leica TCS SP8 or Leica TCS DMi8 confocal microscope and Leica Las X Software; accompanying transmitted light images used Dodt gradient contrast or differential interference contrast imaging, respectively. All fluorescent images were captured through an HC PL APO 63× (numerical aperture 1.3). *Z*-stacks were collected with 0.33 μm slices, with a 600 or 1000 Hz scanning speed and a pinhole setting of 1 AU. SfGFP fusions were excited via a 488 nm laser and emissions between 493 and 547 nm were detected using a HyD sensor. mCherry fusion proteins were excited by a 552 nm laser and emissions between 583 and 784 nm were detected by a HyD sensor. Laser power (1-3%), line accumulation (1-6×) and gain settings (15-75%) varied based on fusion protein. Offset settings were not used. Worms were immobilized using 10 mM levamisole in M9 buffer and mounted on 2% agarose pads supplemented with 2.5% sodium azide. Embryos were staged based on minutes of development past 1.5-fold (Birnbaum et al., 2023). L4 larvae were staged based on vulva morphology (Cohen et al., 2020a; Mok et al., 2015).

### Image analysis

Images were processed using ImageJ (Schindelin et al., 2012). Mander's coefficients of overlap between LPR-1::SfGFP and mCherry::LPR-3 were obtained using the JaCoP plugin and a single confocal *z*-slice from each L4.7 stage animal. Threshold settings were 51 for LPR-1::SfGFP and 43 for mCherry::LPR-3. Peak intensities of fusion proteins in the excretory duct/pore lumen were assessed using the Plot Profile tool and a 10-pixel-wide line drawn across the lumen within maximum projections of five confocal *z*-slices per embryo. Mean gray values of extra-embryonic fusion proteins were assessed using the Measure tool and 2 µm^2^ boxes drawn within a single confocal *z*-slice per embryo. LPR-1::SfGFP puncta number in L4 animals was assessed using the Analyze Puncta tool and a threshold setting of 40. Graphs were generated using Prism GraphPad^®^, where each individual dot represents a single animal, and error bars correspond to the standard deviation. All quantitative image data were compared between genotypes using a non-parametric Mann–Whitney *U*-test.

### Western blots

Western blots with mixed stage LPR-1::SfGFP animals (strain UP3665) were performed as in Cohen et al. (2020b) using primary antibody goat anti-GFP (Rockland Immunochemicals, 600-101-215, 1:1000) and secondary antibody anti-goat-HRP (Rockland Immunochemicals, 605-4302, 1:10,000).

### Lipidomics using liquid chromatography and mass spectrometry

Lipidomics was performed on 1.5-fold-stage embryos from strains N2 (WT), UP2486 [*lpr-1(cs207)*] and UP3167 [*lpr-1(cs207); scav-2(ok877)*], grown in parallel on 9 cm NGM plates seeded with a thick lawn of OP50. For each strain, three to five biological replicates were collected across three independent experiments conducted on different days. Eggs were purified from well-fed day 1/day 2 adults as previously described (Njume et al., 2022). Briefly, eggs prepared by bleaching the adults were washed three times with M9 buffer before being resuspended in 6 ml M9 and gently shaken for 5 h at 20°C to allow development to the 1.5-fold stage. Embryos were purified in a 30% sucrose gradient, washed in Milli-Q water, and the pellet was snap-frozen in 2 ml tubes in liquid nitrogen. Samples were stored at −80°C until further processing. For homogenization, frozen samples were thawed on ice, and a mixture of 0.7 mm zirconia beads (BioSpec Products, 11079107ZX) and polar solvent (500 µl Milli-Q water and 500 µl methanol) was added. Samples were then homogenized using a bullet blender for 5 min at 30 Hz. Subsequently, chloroform was added to reach a final volume of 1 ml. Samples were thoroughly mixed and centrifuged for 10 min at 20,000 ***g*** to induce phase separation. After centrifugation, the apolar (chloroform) phase was collected for lipidomic analysis. This apolar phase was then dried under nitrogen gas and reconstituted in 100 µl chloroform/methanol (2:1, v/v) before injection. Reverse-phase liquid chromatography was performed using a Zorbax XDB C18 column (50×4.6 mm, 1.8 µm) maintained at 40°C. The mobile phases consisted of 5 mM ammonium acetate buffer at pH 5 (solvent A) and isopropanol (solvent B). A gradient elution was applied as follows: 0 min, 90:10 (A:B); 10 min, 20:80; 25 min, 20:80; 27 min, 90:10; 30 min, 90:10, at a flow rate of 0.5 ml/min. Mass spectrometry was carried out on an Agilent QTOF 6520 instrument in autoMSMS mode with a fixed collision energy of 25 eV. Source parameters were: drying gas temperature 350°C, gas flow 7 l/min, nebulizer pressure 50 psi, capillary voltage 4500 V, and fragmentor voltage 210 V. Mass spectrometry (MS) and MS/MS spectra were recorded over an m/z range of 100-1700. The acquired MS data underwent a rigorous analysis, encompassing principal component analysis (PCA), partial least squares discriminant analysis (PLS-DA), heatmaps and univariate analysis. None of these analyses reported consistent differences between genotypes.

Subsequently, the obtained Agilent format ‘.d’ data were converted to .mzXML format using ProteoWizard MSConvert software (v. 3.03.9393, 64-bit) with the peak picking filter option. Preprocessing, which included peak detection, identification, grouping and smoothing, retention time correction, filtration, integration and signal drift correction, was conducted using the freely available Galaxy Workflow4Metabolomics (W4M) platform.

### Dietary lipid supplementation experiments

For dietary lipid supplementation experiments, 5% stock solutions of egg phosphatidylcholine (Sigma-Aldrich, P3556), lyso-phosphatidylcholine (Sigma-Aldrich, L4129) or phosphatidylethanolamine (Sigma-Aldrich, P7943) were made with 100% ethanol and stored at −20°C. Lipid stocks were freshly diluted to 0.01% in M9 buffer just before use. For each lipid, 200 μl was applied to NGM plates and allowed to dry for several hours before seeding with normal OP50 bacteria. For cholesterol experiments, NGM plates were prepared without cholesterol or with 5× the normal amount of cholesterol. For *lpr-1* survival tests, adult P0 hermaphrodites were placed on lipid plates, F1 survivors were used for synchronized egg-lays (still on lipid plates), and F2 progeny were scored for viability. For *pcyt-1* fertility tests, adult P0 hermaphrodites were placed on lipid plates, F1 progeny were transferred as L4s to 25°C and F2 progeny were counted to assess fertility.

## Supplementary Material

10.1242/develop.205309_sup1 Supplementary information

Table S3. Source data for Fig. 6C

Table S4. Source data for Fig. S1

Table S5. Source data for Fig. S3

Table S6. Source data for Fig. S4

Table S7. Source data for Fig. S6

Table S8. Source data for Fig. 1

Table S9. Source data for Fig. 2

Table S10. Source data for Fig. 3

Table S11. Source data for Fig. 4
