# Supplementary material for: Opposing roles for lipocalins and a CD36 family scavenger receptor in apical extracellular matrix-dependent protection of narrow tube integrity
Source: Development. 2026 Jan 12;153(16):dev205309. doi: 10.1242/dev.205309 (PMC12848578; doi:10.1242/dev.205309)
Supplement: Supplementary information [file develop-153-205309-s1.pdf]

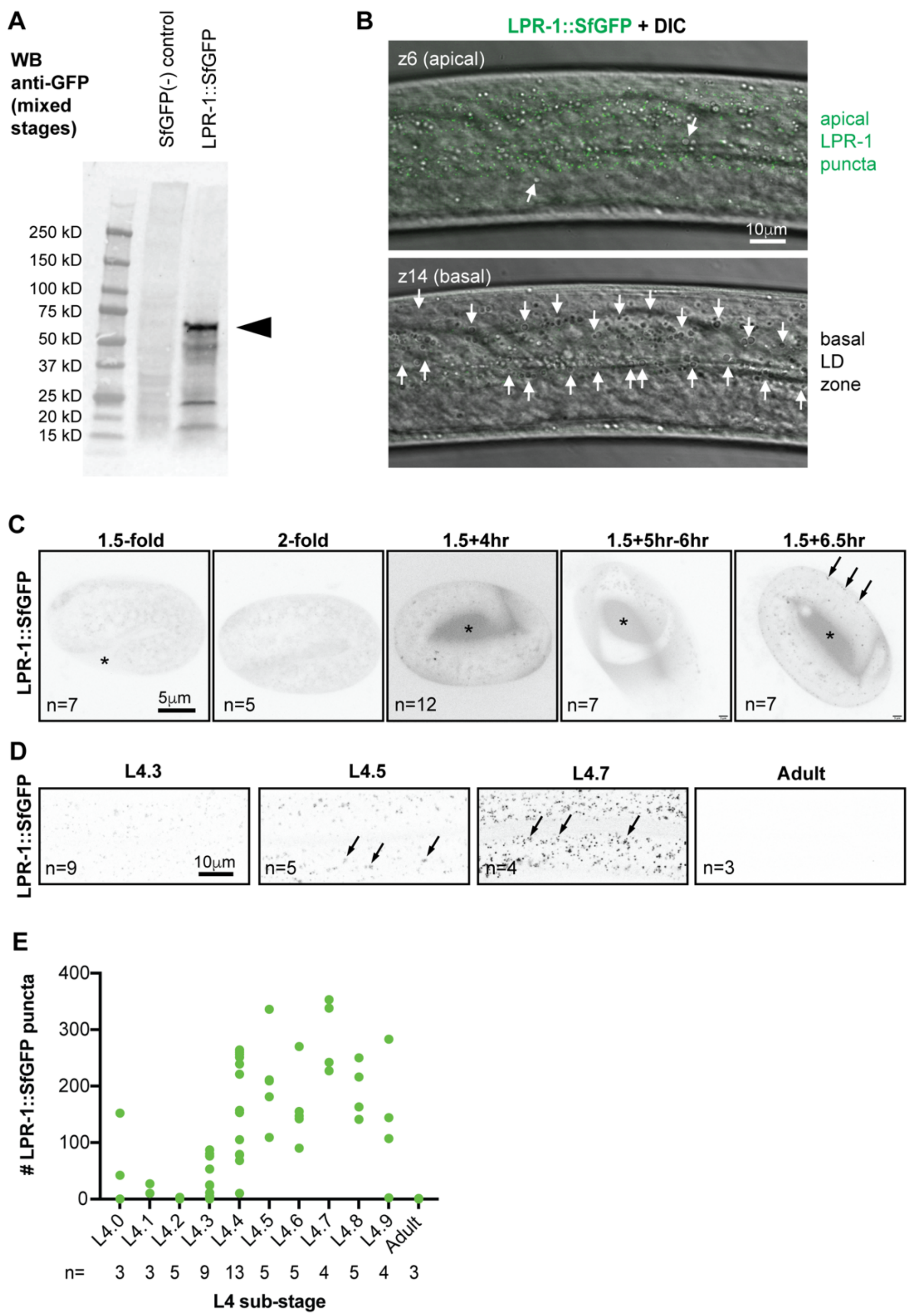

Fig. S1. LPR-1::SfGFP marks apical puncta and the extraembryonic space
A. Western blot of mixed-stage worm lysates probed with anti-GFP antibody. The predominant band (arrowhead) matches the expected 57kD size of full-length tagged LPR-1. Image is representative of two independent blots. B. Confocal slices of the epidermis in a mid-L4 animal. LPR-1::SfGFP puncta are concentrated apically, whereas most lipid droplets (arrows) are concentrated basally within the tissue. C. Timeseries of embryonic development, showing LPR-1::SfGFP accumulating within the extraembryonic space (asterisk) and visible in some puncta (arrows). D. Timeseries of L4 larval development, showing LPR-1::SfGFP puncta (arrows). E. Quantification of puncta number across L4 stages. Puncta were counted within 61 X 38.5 micron single confocal Z-slice images of the mid-body, as shown in D.

Development • Supplementary information

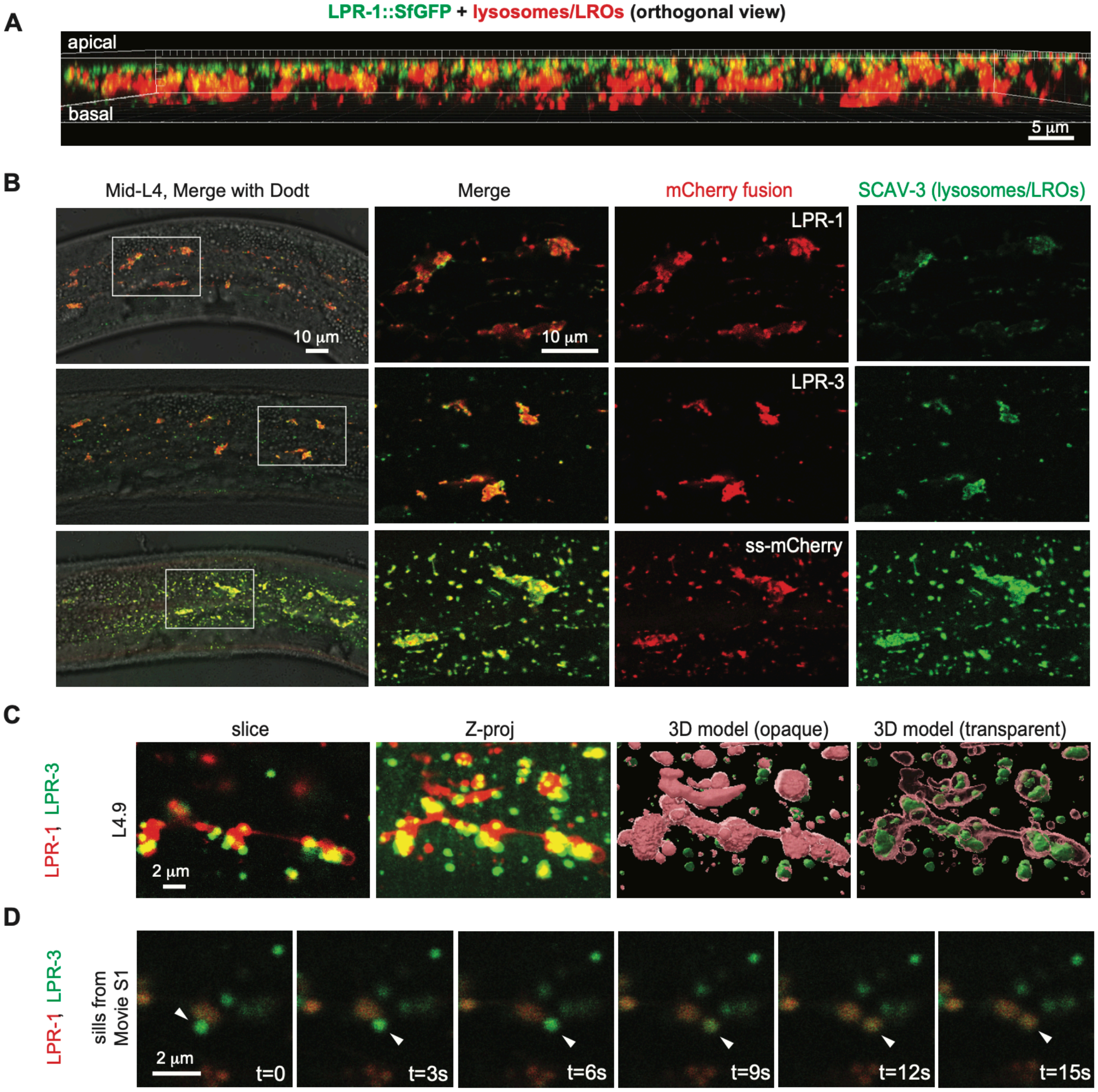

Fig. S2. LPR-1, LPR-3, and control secreted mCherry fusions accumulate in epidermal lysosomes

A. Orthogonal (X-Z) view of confocal Z-stack, showing apical LPR-1::SfGFP puncta (green) relative to NUC-1::mCherry-marked lysosomes (red). Note that larger lysosomes are located basally in the epidermis. B. mCherry-tagged lipocalins and control fusions (red) accumulate strongly within epidermal lysosomes marked by SCAV-3::SfGFP (green). Confocal Z-slices, with boxed regions shown magnified at right. C. SfGFP::LPR-3 puncta cluster near lysosome-like structures marked by LPR-1::mCherry. 3D models were generated using Imaris software (Oxford instruments, UK). D. Stills from Movie S1, showing SfGFP::LPR-3 puncta fusing with a lysosome-like structure marked by LPR-1::mCherry. All images are representative of at least n=10 specimens imaged, except panel D which is representative of 3 movies.

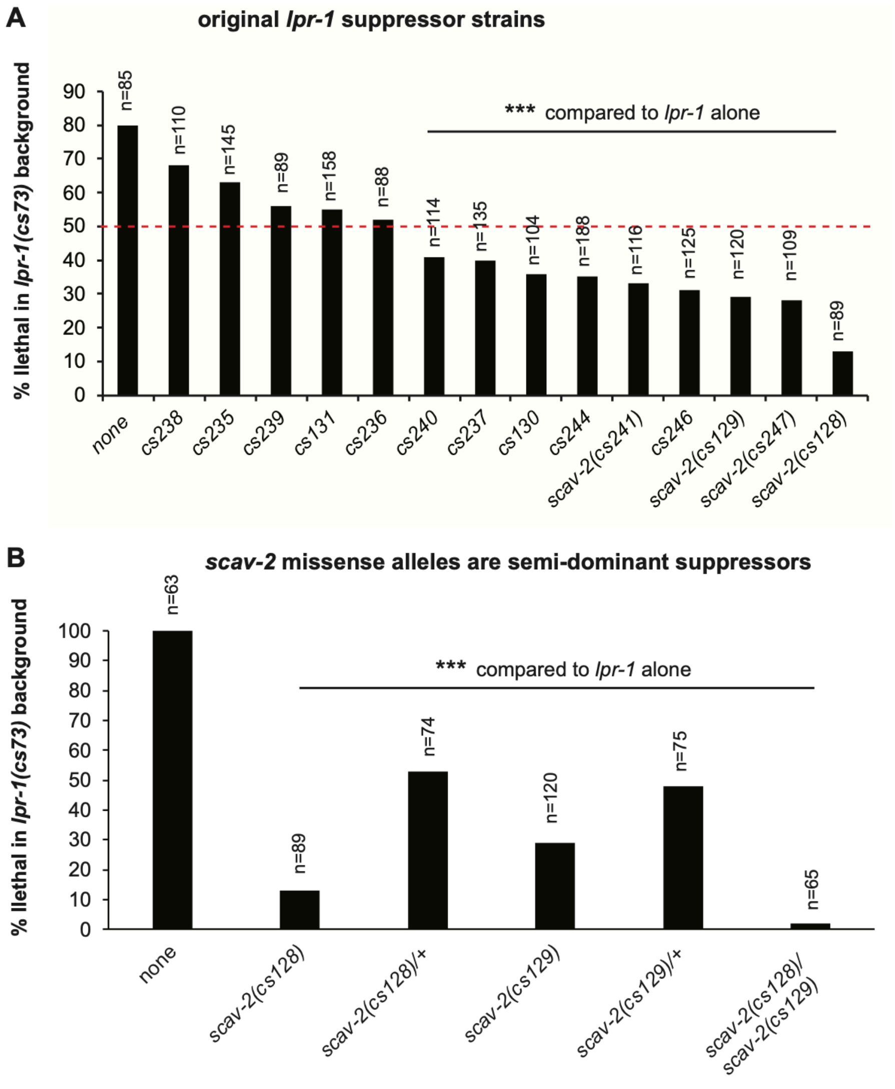

C

Allele	DNA change	Protein change	First obtained as
scav-2(cs128)	*ccg to tcg*	P353S	*lpr-1* suppressor
scav-2(cs129)	*ccg to tcg*	P385S	*lpr-1* suppressor
scav-2(cs230)	*gga to gaa*	G349E	*let-653* suppressor
scav-2(cs241)	*gaa to aaa*	E282K	*lpr-1* suppressor
scav-2(cs247)	*aca to ata*	T343I	*lpr-1* suppressor
scav-2(ok877)	2533 bp deletion/STOP		deletion project allele

Fig. S3. *lpr-1* suppressor alleles

A. Quantification of survival in original *lpr-1* suppressor strains. ***, $p<0.0001$, Fisher's Exact Test, compared to parental strain. Four of the nine top suppressors contained *scav-2* alleles. B. Two *scav-2* alleles tested are each semi-dominant suppressors of *lpr-1* lethality. The data for the homozygous mutants is identical to that in A. C. Molecular lesions in *scav-2* alleles.

Development • Supplementary information

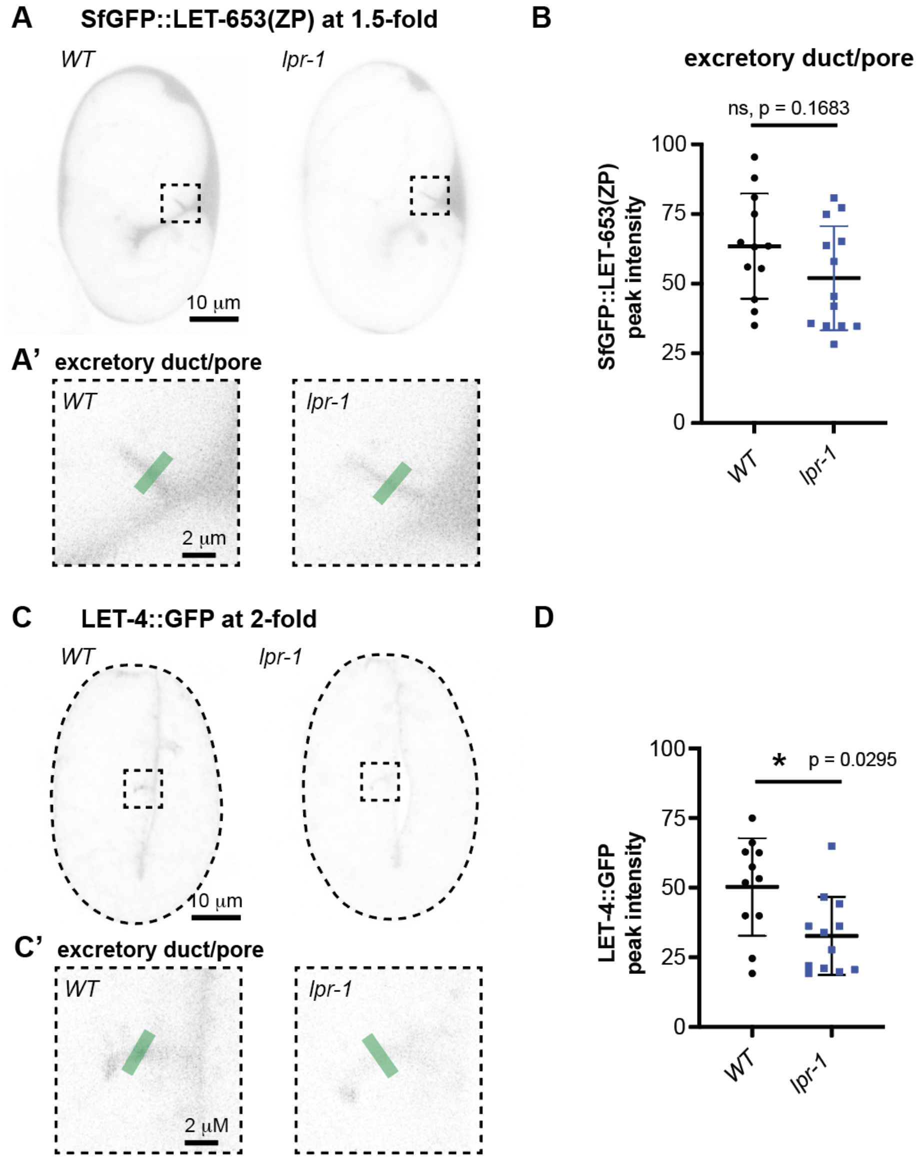

Fig. S4. *lpr-1* loss has little or no effect on excretory duct/pore matrix accumulation of LET-653 and LET-4 fusions

A,B. Loss of *lpr-1* does not substantively impact LET-653(ZP) accumulation within the developing duct and pore tubes. A. Inverted projections of five confocal z-slices from 1.5-fold embryos. Boxed region is shown magnified in A'. Green line indicates duct/pore lumen region analyzed in B. B. Peak intensities of LET-653(ZP)::SfGFP (*csIs66*) duct/pore signal as assessed using the Plot Profile tool in FIJI. ns, Mann-Whitney U test. C-D. Loss of *lpr-1* may slightly decrease LET-4::GFP accumulation within the developing duct and pore tubes. C. Inverted projections of five confocal z-slices from 2-fold embryos. Boxed region is shown magnified in C'. Green line indicates duct/pore lumen region analyzed in D. D. Peak intensities of LET-4::GFP duct/pore signal as assessed using the Plot Profile tool in FIJI. *, p= 0.0295, Mann-Whitney U test.

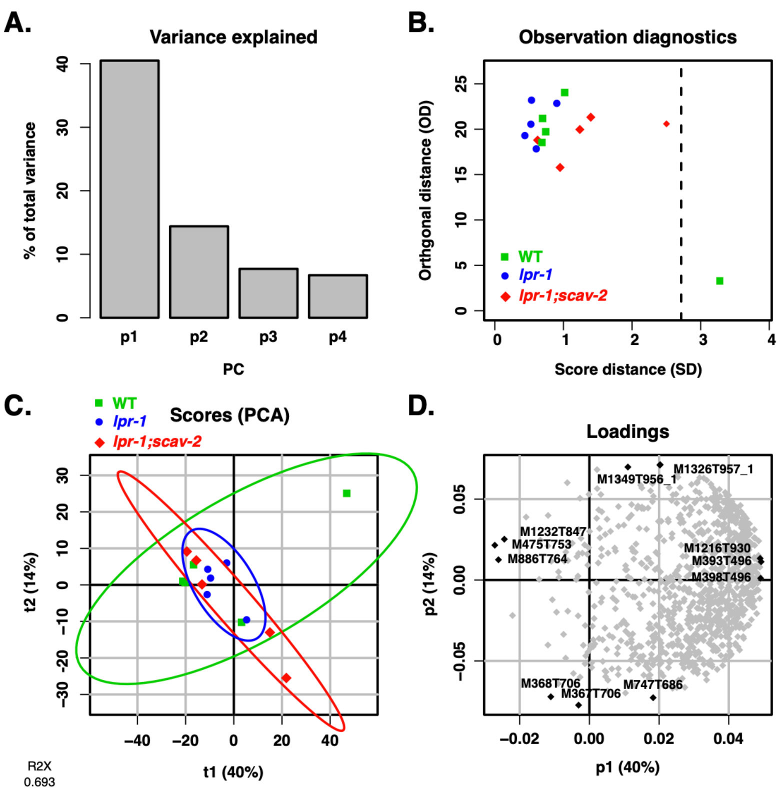

Fig. S5. *lpr-1* mutants do not have large changes in lipid abundance

A. Principal Component Analysis (PCA) of *C. elegans* 1.5-fold embryo samples based on positive ion mode LC-MS data. The first two dimensions explain 40% and 14% of variance. B. A plot of score distance vs. orthogonal distance was used to visualize outliers. One biological sample was removed from the analysis. C. Wild-type animals (N2, green), single mutant (*lpr-1(cs207)*, blue), and double mutant (*lpr-1(cs207); scav-2(ok877)*, red) showed no consistent separation between genotypes across principal components t1 and t2, indicating no robust genotype-specific differences in overall lipid content. **D**. The most representative lipid features contributing to the principal components are visualized in the PCA loading plot.

Development • Supplementary information

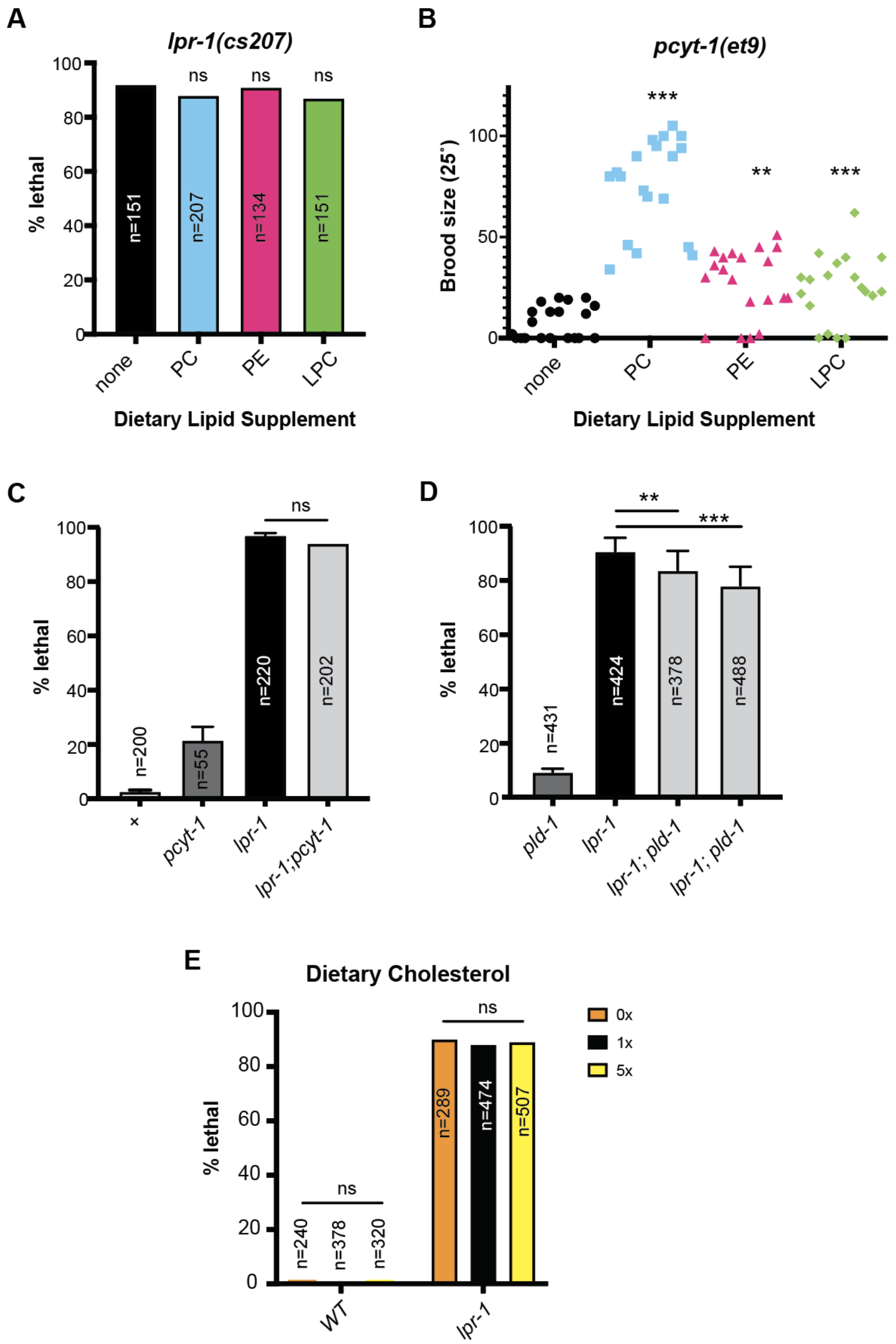
A
lpr-1(cs207)
% lethal
ns
ns
ns
n=151
n=207
n=134
n=151
none
PC
PE
LPC
Dietary Lipid Supplement
B
pcyt-1(et9)
Brood size (25°)
***
**
***
none
PC
PE
LPC
Dietary Lipid Supplement
C
% lethal
ns
n=200
n=55
n=220
n=202
+
pcyt-1
lpr-1
lpr-1;pcyt-1
D
% lethal
**
***
n=431
n=424
n=378
n=488
pld-1
lpr-1
lpr-1; pld-1
lpr-1; pld-1
E
Dietary Cholesterol
% lethal
ns
ns
n=240
n=378
n=320
n=289
n=474
n=507
0x
1x
5x
WT
lpr-1

Fig. S6. Dietary phospholipid or cholesterol manipulations don't affect *lpr-1* mutant survival

A. Dietary phospholipids phosphatidylcholine (PC), phosphatidylethanolamine (PE), and lysophoshatidylcholine (LPC) did not improve *lpr-1* survival. B. Dietary phospholipids did improve fertility of a mutant in *pcyt-1*, which encodes the *C. elegans* ortholog of phosphocholine cytidyltransferase (CCT), the rate-limiting enzyme in both the Kennedy (*de novo*) and phosphatidylethanolamine methyl transferase (PEMT) pathways for PC biosynthesis [93]. C. *pcyt-1(et9)* did not affect *lpr-1* survival. D. A null mutation in Phospholipase D, *pld-1(ok2222),* slightly improved *lpr-1* survival. The effect was seen in two independent lines generated. E. Neither increasing nor decreasing dietary cholesterol affected *lpr-1* survival. Panels show pooled data from one (A,E), two (B,C) or three (D) experimental trials, with each trial containing broods of at least six worms. All data analyzed by Fisher's Exact Test relative to *lpr-1* control, except B analyzed by Mann-Whitney U Test relative to *pcyt-1* control. **p<0.001, ***p<0.0001, ns p>0.05.

Development • Supplementary information

Table S1. Strains used

Strain	Genotype	Source
N2	*wild type*	
ML2578	*let-4(mc95 [LET-4::GFP]) X*	Vuong-Brender et al 2017
PHX7064	*grl-2(syb7064 [SfGFP::GRL-2]) V*	Serra et al 2024
RB973	*scav-2(ok877) III*	CGC
RB1737	*pld-1(ok2222) II*	CGC
UP1787	*let-4(mn105)x; csEx173(C44H4, sur-5::gfp)*	Mancuso et al 2012
UP2436	*lpr-1(cs207) I*	Pu et al 2017
UP3167	*lpr-1(cs207) I; scav-2(ok877) III*	This work
UP3205	*csEx683 [grl-2pro::scav-2(cDNA), lin-48::mRFP, RDY-2::GFP]*	This work
UP3210	*lpr-1(cs207) I; scav-2(ok877) III; csEx683 [grl-2pro::scav-2(cDNA), lin-48::mRFP, RDY-2::GFP]*	This work
UP3244	*let-653(cs178); csEx358[lpr-1p::LET-653b, unc-119p::GFP]*	Gill et al 2016
UP3261	*scav-2(ok877)III; lpr-3(cs144) X; csEx436[lpr-3 fosmid+myo-2::mCherry]*	This work
UP3330	*csEx757[scav-2pro(5.8k)::NLS::GFP;grl-2pro::mRFP]*	This work
UP3422	*csIs66 [let-653pro::SfGFP::LET-653(ZP), let-653pro::PH-mCherry, lin-48pro::mRFP] X*	Gill et al 2016
UP3435	*+/hT2[qIs48] (I;III)*	This work
UP3440	*scav-2(ok877) III; let-4(mn105) IV*	This work
UP3561	*pcyt-1(et9) X*	This work
UP3612	*lpr-1(cs249 [lpr-1::SfGFP]) I original version*	This work
UP3648	*lpr-1(cs249 [lpr-1::SfGFP]) I outcrossed version*	This work
UP3657	*scav-2(ok877) I; let-653(cs178) IV*	This work
UP3665	*lpr-1(cs249 [lpr-1::SfGFP])) I; qxIs257[ced-1pro::NUC-1::mCherry]*	This work
UP3666	*lpr-3(cs250 [SfGFP::lpr-3] X*	Cohen et al 2020
UP3697	*lpr-1(cs251 [lpr-1::mCherry]) I*	This work
UP3711	*lpr-1(cs251 [lpr-1::mCherry]) I; lpr-3(cs250 [SfGFP::lpr-3] X*	This work
UP3745	*lpr-1(cs251 [lpr-1::mCherry]) I; qxIs630 [scav-3::GFP]*	This work
UP3746	*let-653(cs262 [LET-653::SfGFP]) IV*	Cohen et al 2020
UP3808	*lpr-3(cs266[mCherry::lpr-3]) X*	This work
UP3820	*csIs87 [lpr-1pro::ss-mCherry] X; qxIs630 [scav-3::SfGFP]*	This work
UP3823	*lpr-3(cs266 [mCherry::lpr-3] X; qxIs630 [scav-3::SfGFP]*	This work

UP3862	*lpr-1(cs207)/hT2[qIs48] I; lpr-3(cs250 [SfGFP::lpr-3] X*	This work
UP3957	*lpr-1(cs249 [lpr-1::SfGFP]) I; lpr-3(cs266 [mCherry::lpr-3]) X*	This work
UP3986	*scav-2(cs289 [scav-2::SfGFP]) III*	This work
UP4002	*lpr-1(cs207)/hT2[qIs48]; scav-2 (cs289 [scav-2::SfGFP])/hT2 III*	This work
UP4301	*lpr-1(cs207) I; pld-1(ok2222) II*	This work
UP4302	*lpr-1(cs207) I; pld-1(ok2222) II*	This work
UP4354	*lpr-1(cs207) I/ hT2[qIs48] (I;III); pcyt-1(et9) X*	This work
UP4374	*lpr-1(cs207)/hT2[qIs48] I; scav-2(ok877)/hT2 III; lpr-3(cs250 [SfGFP::lpr-3] X*	This work
UP4384	*lpr-1(cs207)/hT2[qIs48] I; scav-2(ok877)/hT2 III; let-653(cs262 [LET-653::SfGFP]) IV*	This work
UP4386	*lpr-1(cs207)/hT2[qIs48] (I;III); grl-2(syb7064 [SfGFP::GRL-2]) V*	This work
UP4387	*lpr-1(cs207) I; scav-2(ok877) III; grl-2(syb7064 [SfGFP::GRL-2]) V*	This work
UP4391	*lpr-1(cs207)/hT2[qIs48] (I;III); let-4(mc95 [LET-4::GFP]) X*	This work
UP4392	*lpr-1(cs207)/hT2[qIs48] (I;III); let-653(cs262 [LET-653::SfGFP]) IV*	This work
UP4395	*scav-2(ok877) III; lpr-3(cs250 [SfGFP::lpr-3] X*	This work
UP4400	*lpr-1(cs207)/hT2[qIs48] (I;III); csIs66 [let-653pro::SfGFP::LET-653(ZP), let-653pro::PH-mCherry, lin-48pro::mRFP] X*	This work
UP4409	*let-653(cs178) IV; lpr-3[cs250 [SfGFP::LPR-3]) X; csEx358[lpr-1p::LET-653b, unc-119p::GFP]*	This work
UP4410	*scav-2(ok877) III; let-653(cs178) IV; lpr-3[cs250 [SfGFP::LPR-3]) X*	This work
XW5399	*qxIs257 [ced-1pro::nuc-1::mCherry]*	Miao et al 2020
XW8056	*qxIs630 [scav-3::GFP]*	Miao et al 2020

Development • Supplementary information

Table S2. Plasmids, Primers, and guide RNAs

Plasmids	Description
pCW11	source of SfGFP
pDD162	eft-3pro::Cas9 + U6pro:empty sgRNA
pJC39	SfGFP^SEC^3xFLAG
pJJR83	mCherry^SEC^3xFLAG
pEP75	grl-2pro::scav-2(cDNA)
pEP88	scav-2pro::NLS-GFP
pPD49.26	expression plasmid backbone
pPD122.13	source of NLS-GFP
pRFR66	eft-3pro::Cas9 + U6pro::lpr-1sgRNA (5'-gatacggcagagtttttgac-3')
pRFR67	lpr-1^SEC^SfGFP repair plasmid
pRFR68	lpr-1^SEC^mCherry repair plasmid

Primers	Sequence
oAB4 (R)	aaaaatatatataactctctactttttttaatttgttaaaactgcaaaaattttcaaaatcaactcaaattatcgatttttctgcaattttcaaaaattcaaggatctaattagctataTCATTTGTAGAGCTCATCCATG
oAB5 (F)	AGCAAAGGAGAAGAACTTTTCAC
oAB6 (R)	TTTGTAGAGCTCATCCATGCC
oAB10 (F)	TCTGTGACCCGATTCTCGGACGGCGGTCAAAACGGGCAAATGGATTGTCACCGGAACCTCTTCCTCCGTATGTTCCGAAGGGAGAACCAAACGGATATCACCATAATCATGCCTACATGAGCAAAGGAGAAGAACTTTTC
oEP89 (F)	GACGGCTAGCAAAATGCCTTCCAGCAATAATTCG
oEP90 (R)	GGGAAAGAGCTCTCACATGTAGGCATGATTATGG
oEP92 (R)	gggaaGTCGACGAATAAAGCTTCTGTTCTCTGTCC
oEP96 (F)	gggaaGCATGCCCGTGCTAAATCCGAAGTATTAC

crRNAs	Sequence
crAB1	ACATGTAGGCATGATTATGG
crAB2	TAATTAGCTATATCACATGT

Development • Supplementary information

Table S3. Source data for Fig. 6C

Available for download at
https://journals.biologists.com/dev/article-lookup/doi/10.1242/dev.205309#supplementary-data

Table S4. Source data for Fig. S1

Available for download at
https://journals.biologists.com/dev/article-lookup/doi/10.1242/dev.205309#supplementary-data

Table S5. Source data for Fig. S3

Available for download at
https://journals.biologists.com/dev/article-lookup/doi/10.1242/dev.205309#supplementary-data

Table S6. Source data for Fig. S4

Available for download at
https://journals.biologists.com/dev/article-lookup/doi/10.1242/dev.205309#supplementary-data

Table S7. Source data for Fig. S6

Available for download at
https://journals.biologists.com/dev/article-lookup/doi/10.1242/dev.205309#supplementary-data

Table S8. Source data for Fig. 1

Available for download at
https://journals.biologists.com/dev/article-lookup/doi/10.1242/dev.205309#supplementary-data

Table S9. Source data for Fig. 2

Available for download at
https://journals.biologists.com/dev/article-lookup/doi/10.1242/dev.205309#supplementary-data

Table S10. Source data for Fig. 3

Available for download at
https://journals.biologists.com/dev/article-lookup/doi/10.1242/dev.205309#supplementary-data

Table S11. Source data for Fig. 4

Available for download at
https://journals.biologists.com/dev/article-lookup/doi/10.1242/dev.205309#supplementary-data

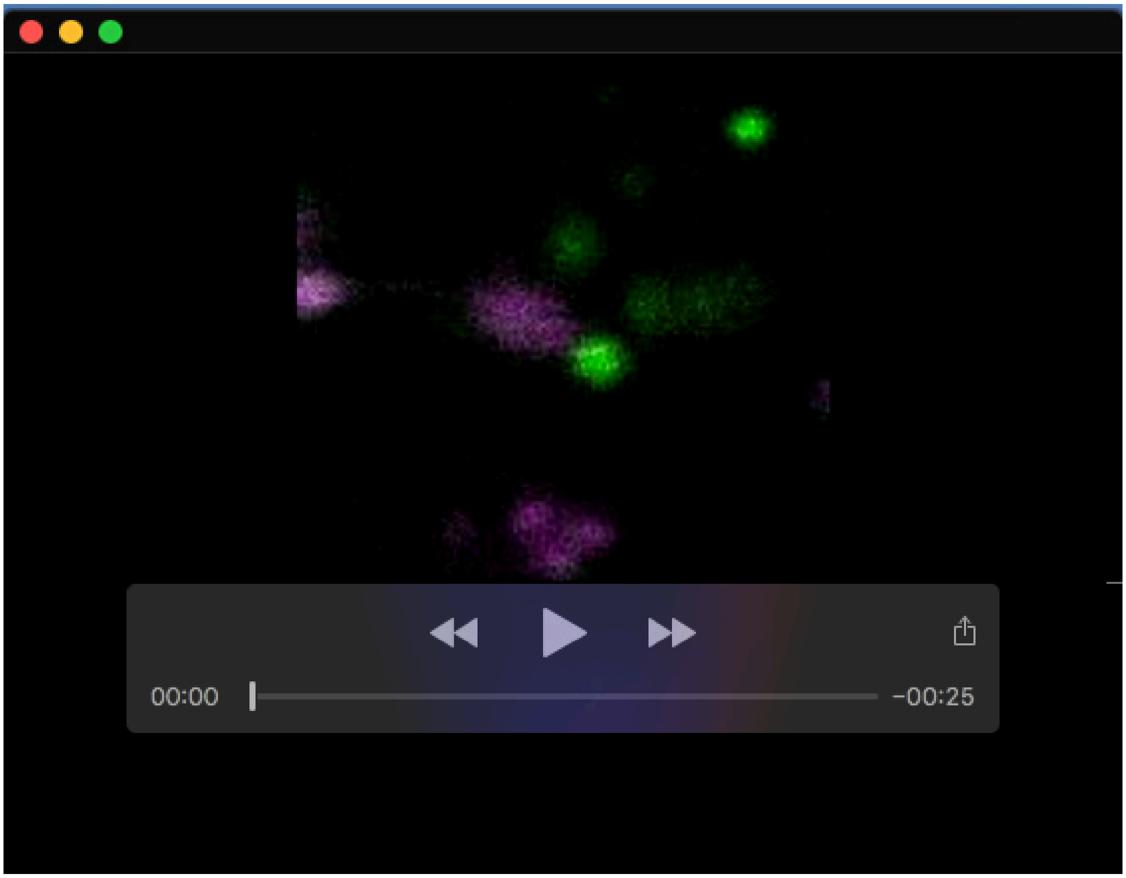

Movie 1. SfGFP::LPR-3 puncta fusing with a lysosome-like structure marked by LPR- 1::mCherry
